# Deploying QTL-seq for rapid delineation of a potential candidate gene underlying major trait-associated QTL in chickpea

**DOI:** 10.1093/dnares/dsv004

**Published:** 2015-04-27

**Authors:** Shouvik Das, Hari D. Upadhyaya, Deepak Bajaj, Alice Kujur, Saurabh Badoni, Laxmi, Vinod Kumar, Shailesh Tripathi, C. L. Laxmipathi Gowda, Shivali Sharma, Sube Singh, Akhilesh K. Tyagi, Swarup K. Parida

**Affiliations:** 1 National Institute of Plant Genome Research (NIPGR), New Delhi 110067, India; 2 International Crops Research Institute for the Semi-Arid Tropics (ICRISAT), Patancheru, Telangana 502324, India; 3 National Research Centre on Plant Biotechnology (NRCPB), New Delhi 110012, India; 4 Division of Genetics, Indian Agricultural Research Institute (IARI), New Delhi 110012, India

**Keywords:** chickpea, NGS, QTL, QTL-seq, SNP

## Abstract

A rapid high-resolution genome-wide strategy for molecular mapping of major QTL(s)/gene(s) regulating important agronomic traits is vital for in-depth dissection of complex quantitative traits and genetic enhancement in chickpea. The present study for the first time employed a NGS-based whole-genome QTL-seq strategy to identify one major genomic region harbouring a robust 100-seed weight QTL using an intra-specific 221 chickpea mapping population (*desi* cv. ICC 7184 × *desi* cv. ICC 15061). The QTL-seq-derived major SW QTL (*CaqSW1.1*) was further validated by single-nucleotide polymorphism (SNP) and simple sequence repeat (SSR) marker-based traditional QTL mapping (47.6% *R*^2^ at higher LOD >19). This reflects the reliability and efficacy of QTL-seq as a strategy for rapid genome-wide scanning and fine mapping of major trait regulatory QTLs in chickpea. The use of QTL-seq and classical QTL mapping in combination narrowed down the 1.37 Mb (comprising 177 genes) major SW QTL (*CaqSW1.1*) region into a 35 kb genomic interval on *desi* chickpea chromosome 1 containing six genes. One coding SNP (G/A)-carrying constitutive photomorphogenic9 (COP9) signalosome complex subunit 8 (*CSN8*) gene of these exhibited seed-specific expression, including pronounced differential up-/down-regulation in low and high seed weight mapping parents and homozygous individuals during seed development. The coding SNP mined in this potential seed weight-governing candidate *CSN8* gene was found to be present exclusively in all cultivated species/genotypes, but not in any wild species/genotypes of primary, secondary and tertiary gene pools. This indicates the effect of strong artificial and/or natural selection pressure on target SW locus during chickpea domestication. The proposed QTL-seq-driven integrated genome-wide strategy has potential to delineate major candidate gene(s) harbouring a robust trait regulatory QTL rapidly with optimal use of resources. This will further assist us to extrapolate the molecular mechanism underlying complex quantitative traits at a genome-wide scale leading to fast-paced marker-assisted genetic improvement in diverse crop plants, including chickpea.

## 1. Introduction

Chickpea is among the most important food legumes of the world. To meet the dietary protein demand of the fast increasing global population, it is vital to raise the world-wide chickpea production and seed/pod yield potential. The seed size/100-seed weight, one of the most crucial seed and pod yield-contributing trait, has always been a trait of consumer preference and trade, besides an important component of domestication and adaptation in chickpea.^1,2^ The enhanced productivity in chickpea can be effectively achieved by developing its high-yielding (increased seed size/weight) durable stress-tolerant improved varieties. Most of these yield-contributing (seed size/weight) and stress tolerance traits targeted for chickpea genetic enhancement are complex and quantitative in nature and governed by multiple major and/or minor genes/QTLs (quantitative trait loci). Identification and fine mapping/map-based cloning of genes underlying QTLs controlling important agronomic traits have been established as the most effective approach for quantitative dissection of these complex traits in crop plants, including chickpea. Significant efforts have been made towards identifying the QTLs associated with diverse yield component and abiotic/biotic stress tolerance traits [including flowering and maturation time, plant growth habit, plant height, seed size/100-seed weight, double podding, seed/pod number per plant and harvest index, nodulation, disease resistance (*Fusarium* wilt, *Ascochyta* blight and *Botrytis* gray mold), and salinity and drought tolerance (root traits)] in chickpea.^3–27^ However, only limited number of these QTLs alongside the QTLs regulating seed weight, nodulation, drought tolerance, and *Fusarium* wilt and *Ascochyta* blight resistance traits have been fine mapped till date and subsequently utilized for marker-assisted genetic improvement of chickpea.^23,24,26–29^ To drive the fine mapping of trait-regulating QTLs, the large-scale genotyping of simple sequence repeat (SSR) and prior discovered single-nucleotide polymorphism (SNP) markers in individuals of inter-/intra-specific mapping populations employing multiple high-throughput genotyping assays are found to be fairly suitable in chickpea.^30–32,24^ This includes SSR markers-based genotyping assay like fluorescent dye-labelled automated fragment analyzer and array-based genotyping platforms, including Illumina GoldenGate/Infinium and Competitive Allele Specific PCR (KASPar) assays and MALDI-TOF (matrix-assisted laser desorption ionization-time of flight) mass array for large-scale genotyping of SNP markers. However, in chickpea with low marker genetic polymorphism, these approaches demand huge cost, labour, time and resources for detecting polymorphic markers between the parents of mapping populations and also sample-by-sample low-throughput genotyping of limited number of markers in a larger set of mapping individuals. Interestingly, the fine mapping of trait-associated QTLs has now become simpler and easy to accomplish with advent of a next-generation sequencing (NGS)-based genotyping-by-sequencing (GBS) assay that demonstrated its efficacy by simultaneous discovery and genotyping of SNPs in mapping individuals at a genome-wide scale.^33–41^ The need of extensive bioinformatics analysis and suitable computational genomics tools in SNP imputation and scanning of high-quality and non-erroneous SNP genotyping information, particularly from individuals of advanced generation mapping populations could restrain the use of GBS assay for molecular mapping of QTLs in preliminary populations of chickpea. Henceforth, efforts should be made to develop an alternative genome-wide strategy that involves less cost, time and labour for high-resolution QTL identification in chickpea.

In this perspective, a high-throughput genome-wide QTL-seq strategy that employs NGS technology for whole-genome resequencing of two DNA bulks of progeny from a segregating mapping population with extremely contrasting phenotypic trait values seems quite pertinent. Since its inception, this has become the most popular approach for quick identification of high-resolution robust and major QTLs controlling qualitative and quantitative traits in crop plants.^42^ Quite recently, this strategy when employed in preliminary F_2_ and advanced generation recombinant inbred line (RIL) mapping populations successfully identified major genes underlying QTLs associated with seedling vigour and blast resistance in rice and flowering time in cucumber.^42,43^ The wider applicability and added advantages of QTL-seq over other traditional QTL mapping strategies available in diverse plant species have been truly realized.^42,43^ Henceforth, its usage in rapid genome-wide scanning and fine mapping of major genes underlying robust QTLs in a large chickpea genome with narrow genetic base assumes relevance. This could eventually accelerate the genomics-assisted breeding as well as genetic improvement of chickpea with optimal use of resources.

As a proof of concept, the present study for the first time made an effort to implement a genome-wide NGS-based high-throughput QTL-seq approach in an intra-specific (*desi*) F_4_ mapping population (ICC 7184 × ICC 15061) for identifying a major genomic region harbouring the robust QTL associated with 100-seed weight in chickpea. The QTL-seq-derived major seed weight QTL was further validated by SNP and SSR marker-based classical QTL mapping. The integration of QTL-seq and traditional QTL mapping with differential expression profiling delineated a potential candidate gene at the major QTL interval regulating seed weight in chickpea. Mining of novel allelic variants and analysis of natural allelic diversity in this seed weight-associated potential gene across diverse cultivated and wild genotypes gave deeper insight into the complex seed weight trait evolution during chickpea domestication.

## 2. Materials and methods

### 2.1. Phenotyping of mapping population for agronomic traits

An intra-specific 221 F_4_ mapping population (ICC 7184 × ICC 15061) derived from low (*desi* cv. ICC 7184 with 100-seed weight 8.9 g) and high (*desi* cv. ICC 15061 with 30.1 g) seed weight landraces was developed by single seed descent method. The mapping individuals and parental genotypes were grown (following α-design field plot method) for three consecutive years (2010–13) with at least two replications during the crop growing season at two diverse geographical locations of India. The mapping individuals along with parental genotypes were phenotyped individually for 100-seed weight (SW). The SW (g) was estimated by considering the average weight (g) of 100-matured seeds at 10% moisture content by selecting 10–12 representative plants from each mapping individuals. The diverse statistical attributes, including coefficient of variation (CV), broad-sense heritability (H^2^), frequency distribution, correlation coefficient and analysis of variance (ANOVA) of SW in mapping population, were analysed using SPSS v17.0 (http://www.spss.com/statistics) and following the methods of Kujur *et al.*^20,21^

### 2.2. NGS-based whole-genome sequencing and QTL-seq analysis

For QTL-seq study, 10 of each low and high seed weight homozygous mapping individuals representing two extreme ends of SW normal frequency distribution curve were screened based on the preliminary clues obtained from our conventional QTL mapping study. The homozygous genetic constitution of these individuals for either of the low and high seed weight trait was assured in QTL mapping through their multi-location/years replicated field phenotyping as well as genotyping of genome-wide well-distributed 96 SSR markers*.*^20,21^ The high-quality genomic DNA isolated from fresh leaves of these homozygous mapping individuals was quantified (Qubit 2.0 Fluorometer, Life Technologies, USA) to equal concentration. The isolated genomic DNA from 10 of each low and high seed weight homozygous mapping individuals were pooled together with an equal ratio (amount) to constitute LSB (low seed weight bulk) and HSB (high seed weight bulk), respectively. About 5–10 µg of DNA isolated from two bulked samples and two parental genotypes were used to construct pair-end sequencing libraries (100-bp read length). These libraries were sequenced individually using HiSeq2000 (Illumina Inc., San Diego, CA, USA) NGS platform. The FASTQ raw sequence reads with a minimum *phred* Q-score of 30 across >95% of nucleotide sequences were considered as high quality. These sequences were further rechecked for their quality using FASTQC v0.10.1. The filtered high-quality sequences obtained from two bulks and parental genotypes were aligned and mapped on to the reference *desi* (ICC 4958^44^) chickpea genome using Burrows-Wheeler alignment tool (BWA) with default parameters.^45^ The high-quality SNPs (minimum sequence read depth: 10 with SNP base quality ≥20) were discovered using SAM tools^46^ and following the detail procedures of Lu *et al.*^43^ and Takagi *et al.*^42^

A well-documented QTL-seq approach relying on the estimation of SNP-index and Δ (SNP-index) (following the recommended parameters of Abe *et al.*^47^; Takagi *et al.*^42^; Lu *et al.*^43^) were used to identify candidate genomic region(s) harbouring the major QTL(s) associated with seed weight in chickpea. In our study, Δ (SNP-index) was measured based on subtraction of SNP-index (proportion of sequence reads supported the SNPs which are altogether different from the reference *desi* genome sequences) between LSW and HSW bulks. The SNP-index was measured as ‘0’ and ‘1’, when entire short sequence reads contained genomic fragments derived from ICC 7184 and ICC 15061, respectively. An average distribution of Δ (SNP-index) of SNPs physically mapped across eight *desi* chromosomes was estimated in a given genomic interval by using sliding window approach with 5 Mb window size and 10 kb increment. The Δ (SNP-index) of LSB and HSB and their corresponding SNP-index within the specified window size were plotted in a graph to generate SNP-index plots. To improve the accuracy of QTL identification through QTL-seq, we estimated the statistical confidence intervals of Δ (SNP-index) with a given read depth under the null hypothesis of no QTLs, following the detail procedures of Takagi *et al.*^42^ and Lu *et al.*^43^

### 2.3. High-throughput genotyping of SNP and SSR markers in an intra-specific mapping population

To validate the major SW QTL identified by QTL-seq, the traditional QTL mapping by selecting the SNP and SSR markers (physically mapped on *desi* chromosome 1 in which major QTL was identified through QTL-seq) showing polymorphism between parental genotypes (ICC 7184 and ICC 15061) was performed. A selected 192 SNPs (physically mapped on *desi* chromosome 1) differentiating ICC 7184 and ICC 15061 were used for their validation and high-throughput genotyping in 192 representative mapping individuals and parental genotypes using Illumina GoldenGate assay. The custom oligo pool assay (OPA) designing, custom Sentrix Array Matrix (SAM) synthesis and GoldenGate SNP genotyping assays (including allele-specific oligonucleotide hybridization, and multiplexed primer extension and ligation reaction) were performed using the genomic DNA of mapping individuals and parental genotypes (following the standard manufacturer's protocol of Illumina, San Diego, CA, USA with minor modifications).^31,48^ The hybridization of fluorescent dye-labelled PCR products onto a decoded SAM was performed using Illumina BeadArray Express Reader. The Illumina GenomeStudio Genotyping software V2011.1 was used for normalization of intensity data and assigning cluster positions of each SNP. To assign valid genotypes at each SNP locus and for measuring the reliability of SNP detection based on distribution of genotypic classes, minimum GenCall and GenTrain cut-off scores of 0.3 were used. The cluster separation score provided by GenCall software module for 192 mapping individuals and parental genotypes was optimized manually based on degree of separation between homozygous and heterozygous clusters as normalized θ value [(2/π) Tan^−1^ (Cy5/Cy3)] in each SNP locus.

Moreover, 48 previously reported genomic SSR markers^49–51^ (physically/genetically mapped on chromosome 1) showing polymorphism between parents of mapping population were selected. The synthesized SSR markers (normal and/or fluorescent dye-labelled) were PCR amplified in the genomic DNA of 192 selected mapping individuals and parental genotypes using touchdown thermal cycling profiling and standard PCR constituents^20,52^ and resolved on 3.5% metaphor agarose gel and automated fragment analyzer. For automated fragment analysis, the amplified FAM-labelled PCR products along with ABI GeneScan-600 LIZ size standard (Applied Biosystems, IL, USA) were resolved in automated 96 capillary ABI 3730xl DNA Analyzer. The electropherogram containing trace files were analysed (as per Kujur *et al.*^20^) using GeneMapper V4.0.

### 2.4. Traditional QTL mapping

The SNP and SSR marker genotyping data showing goodness of fit to the expected Mendelian 1:1 segregation ratio were used for linkage analysis using JoinMap 4.1 (http://www.kyazma.nl/index.php/mc.JoinMap) at higher LOD (logarithm of odds) threshold (>5.0) with Kosambi mapping function. The markers integrated into linkage group (LG) based on their centiMorgan (cM) genetic distance were designated corresponding to genetic/physical positions of markers (anchor SSR markers) mapped on the chromosome as reported by previous studies.^49,50,53–56^ The QTL mapping was performed by integrating the genotyping data of markers mapped on LG of intra-specific genetic map with multi-location/years replicated SW field phenotyping data of 192 mapping individuals. The composite interval mapping function of QTL Cartographer v2.5^57^ and multiple QTL model (MQM mapping) of MapQTL v6.1^58^ at significant (*P* ≤ 0.05) LOD threshold score of ≥4.0 (1,000 permutations) were used to estimate the phenotypic variation explained (PVE, *R*^2^%) by the QTLs and their additive effect on SW trait.

### 2.5. Differential gene expression profiling

To infer the gene regulation patterns during seed development, differential expression profiling using the genes annotated at the major genomic interval underlying a robust SW QTL (identified by both QTL-seq and traditional QTL mapping) was performed. The RNA isolated from different vegetative (leaf) and reproductive (seed) tissues and two seed developmental stages^20,21,24^ of low and high seed weight homozygous mapping individuals (used in QTL-seq analysis) and parental genotypes was amplified using the gene-specific primers via semi-quantitative and quantitative RT–PCR assays (following Kujur *et al.*^20^).

### 2.6. Allele mining and natural allelic diversity in a seed weight-regulating candidate gene

For mining the novel alleles and determining the natural allelic diversity, one SW-associated candidate gene validated by QTL-seq, traditional QTL mapping and differential expression profiling was targeted. The fragments covering the entire coding and non-coding sequence components (including 2 kb upstream and 1 kb downstream regulatory region) of this gene were amplified using the genomic DNA of 94 cultivated and wild chickpea genotypes (belonging to primary, secondary and tertiary gene pools^59^) along with mapping parents and individuals constituting the LSB and HSB. The amplicons were cloned and sequenced, and SNPs were mined within the gene following Kujur *et al.*^20,21^ To estimate the average pair-wise nucleotide diversity (θπ) and Watterson's estimator of segregating sites (θω), the SNP genotyping data were analysed with MEGA v5.0^60^ and TASSEL v3.0 (http://www.maizegenetics.net).

## 3. Results

### 3.1. Genetic inheritance pattern of seed weight in an intra-specific chickpea mapping population

**Figure 1.**
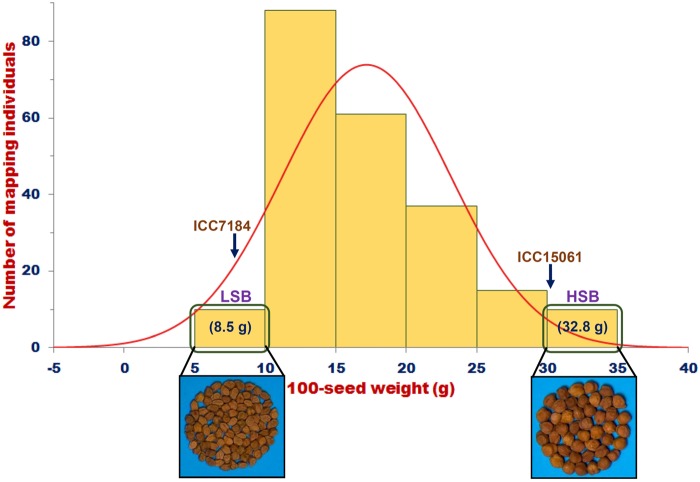
Frequency distribution of 100-seed weight (g) in 221 individuals and parental genotypes of an intra-specific F_4_ mapping population (ICC 7184 × ICC 15061) depicted goodness of fit to the normal distribution. In LSB (low seed weight bulk) and HSB (high seed weight bulk), 100-seed weight varied from 5 to 10 g (mean 8.5 g) and 30 to 35 g (32.8 g), respectively. This figure is available in black and white in print and in colour at *DNA Research* online.

ANOVA indicated a significant difference of SW (7.0–34.9 g with 26–29% CV and 85–88% H^2^) in 221 F_4_ individuals and parental genotypes of an intra-specific mapping population (ICC 7184 × ICC 15061) across 3 yrs (2010–13) (Table 1). The continuous variation as well as normal frequency distribution of SW in mapping individuals and parental genotypes was apparent (Fig. 1). We observed a bi-directional transgressive segregation of SW beyond that of parental genotypes in the mapping population. All these collectively inferred the quantitative genetic inheritance pattern of SW in our developed mapping population of chickpea.

**Table 1.** Statistical measures of 100-seed weight estimated in parental genotypes and 221 individuals of an intra-specific F_4_ mapping population (ICC 7184 × ICC 15061)

Traits	Years^a^	Parental genotypes		F_4_ mapping population			Heritability (%)
		ICC 7184	ICC 15061	Mean ± S.D.	Range	Coefficient of variation (CV%)	
100-seed weight (g) (SW)	2010–11	9.1 ± 2.0	30.7 ± 2.1	17.2 ± 5.0	7.4–34.9	29	85
	2011–12	9.3 ± 2.7	29.5 ± 1.9	16.8 ± 4.3	7.1–33.5	26	88
	2012–13	8.8 ± 2.1	30.2 ± 1.7	16.3 ± 4.7	7.0–33.8	29	87

^a^One-way ANOVA probabilities (F-Prob) at *P* ≤ 0.01 showing significant trait variation in a mapping population across 3 yrs.

### 3.2. Identification and mapping of a major QTL governing seed weight using QTL-seq

**Figure 2.**
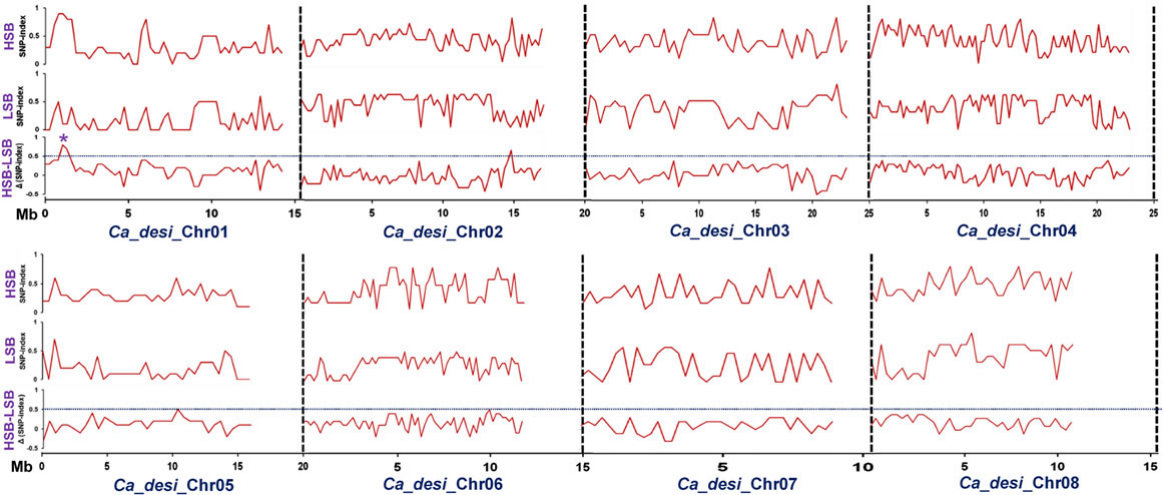
SNP-index graphs depicting the HSB (high seed weight bulk), LSW (low seed weight bulk) and Δ (SNP-index) graphs generated from QTL-seq study. The *X*-axis denotes the physical positions (Mb) of eight *desi* chickpea chromosomes. The *Y*-axis represents the SNP-index, which is estimated according to 5 Mb physical interval with a 10 kb sliding window. Using the statistical confidence intervals under null hypothesis of no QTL (*P* < 0.05) as per Takagi *et al.,*^42^ the Δ (SNP-index) was plotted. One candidate major genomic interval (836,859–872,247 bp) (marked by asterisk) harbouring a robust SW QTL (*CaqSW1.1*) was defined using the criteria of SNP-index near to 1 and 0 in HSB and LSB, respectively, and the confidence value of Δ (SNP-index) above 0.5 (at significance level *P* < 0.05). This figure is available in black and white in print and in colour at *DNA Research* online.

**Figure 3.**
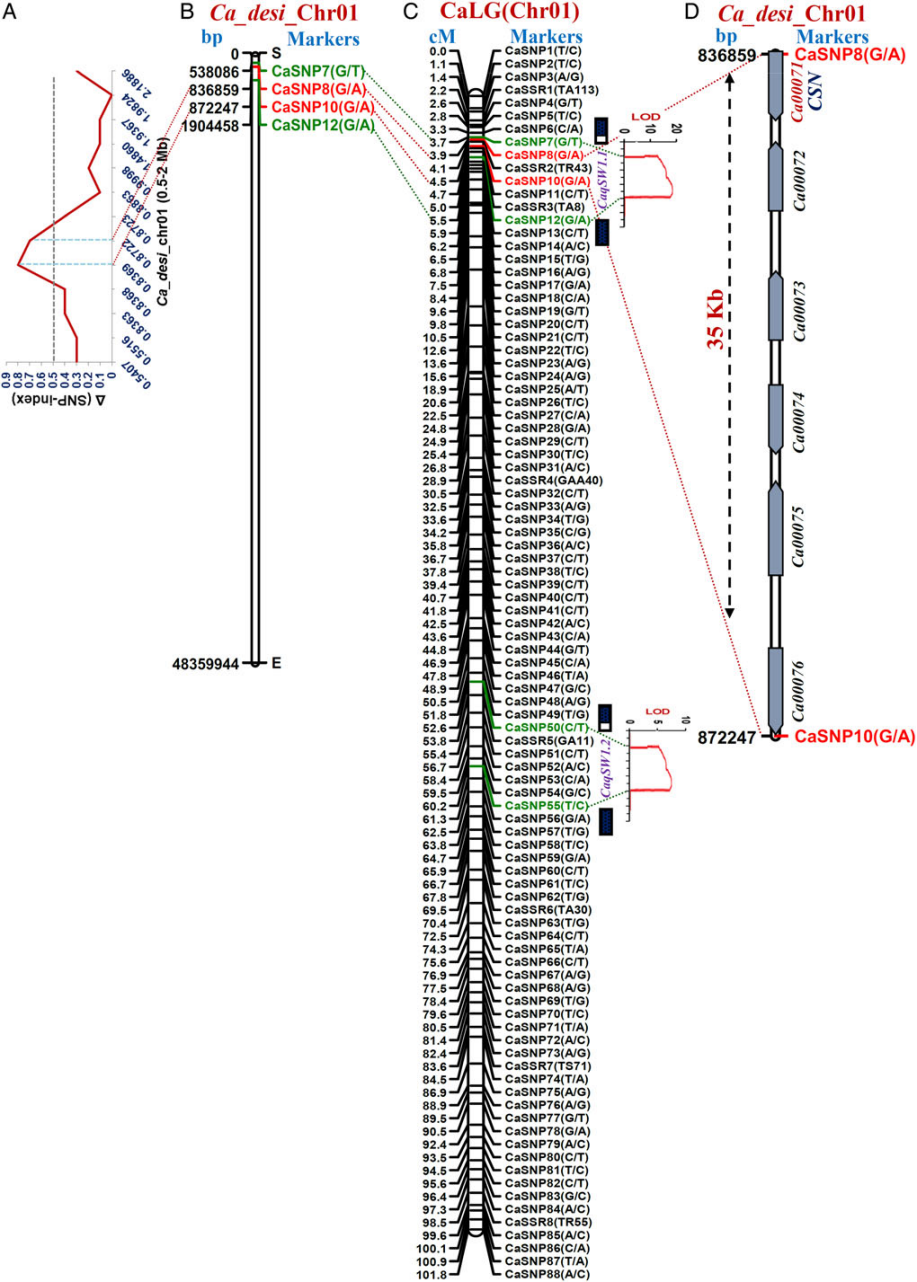
Integration of QTL-seq-derived Δ (SNP-index) graph (A) and *desi* chickpea physical map (B) with traditional QTL mapping (C) identified and mapped one major genomic region underlying a robust SW QTL (*CaqSW1.1*) on 35 kb sequence interval (between marker CaSNP8 and CaSNP10) of chromosome 1. The structural and functional annotation of this target genomic interval was found to contain six protein-coding genes. One coding G/A SNP-carrying *CSN8* gene (tightly linked with *CaqSW1.1*) of these, considered as potential candidate for seed weight regulation in chickpea. The genetic (cM) and/or physical (bp) positions and identity of the markers mapped on the LG/chromosomes are specified on the left and right side of the chromosomes, respectively. The markers flanking/linked with the SW QTL intervals identified by QTL-seq and conventional QTL mapping are marked with red and green fonts/dotted lines, respectively. This figure is available in black and white in print and in colour at *DNA Research* online.

The NGS-based high-throughput whole-genome resequencing of two parental genotypes as well as LSB and HSB of an intra-specific mapping population generated ∼61–65 millions of 100-bp high-quality short sequence reads covering 91–93% of reference *desi* genome. The comparative genome sequence analyses of these parental and bulk samples identified 118,321 high-quality SNPs (with read depth ≥10 and SNP base quality ≥20). The SNP-index of individual SNP differentiating the ICC 7184 and LSB from ICC 15061 and HSB was estimated. The average SNP-index across a 5 Mb genomic interval was measured individually in LSB and HSB using a 10-kb sliding window approach and plotted against all eight chromosomes of *desi* reference genome (Fig. 2). The Δ (SNP-index) was calculated by integrating the SNP-index information of LSB and HSB, and plotted against the genomic positions (Mb) of *desi* genome. Following the principle of SNP-index estimation in QTL-seq analysis,^42,43^ a major genomic region (836,859 to 872,247 bp) on chromosome 1 displaying an average SNP-index of higher than 0.9 in HSB and lower than 0.1 in LSB was identified (Fig. 2). The detailed analysis of this target genomic region indicated that the low and high seed weight mapping individuals constituting the LSB and HSB contained most of the SNP alleles from ICC 7184 and ICC 15061, respectively. Moreover, one major genomic region [CaSNP8 (836,859 bp) to CaSNP10 (872,247 bp)] harbouring a SW QTL identified on chromosome 1 had Δ (SNP-index) value significantly different from 0 at 99% significance level (Figs 2 and 3A and B). These findings by QTL-seq confirmed the presence of a major QTL (designated as *CaqSW1.1*) regulating SW at the 35 kb [836,859 (CaSNP8) to 872,247 (CaSNP10) bp] genomic interval on chromosome 1 of chickpea (Fig. 3A and B). All the SNPs localized at the target SW QTL (*CaqSW1.1*) interval (identified by QTL-seq) with expected allelic discrimination were further validated through resequencing of PCR amplicons amplified from the mapping individuals constituting the LSB and HSB, including parental genotypes.

### 3.3. Validation of QTL-seq-derived seed weight QTL through traditional QTL mapping

To check the accuracy of a major SW QTL identified by QTL-seq, the traditional QTL mapping was performed. For this, the genotyping data of 95 markers (including 87 SNPs and 8 genomic SSR anchor markers mapped on a high-density intra-specific genetic map of LG/chromosome 1) showing polymorphism between parental genotypes as well as LSB and HSB was integrated with multi-location/years replicated field phenotyping data of 192 F_4_ mapping individuals (Supplementary Table S1, Fig. 3C). The interval mapping and composite interval mapping-based classical QTL analysis identified and mapped one major genomic region [CaSNP7 (3.7 cM) to CaSNP12 (5.5 cM)] harbouring a significant robust (LOD: 19.7) SW QTL (*CaqSW1.1*) (covered by seven SNP and SSR markers) on chickpea chromosome 1 (Fig. 3C). The integration of genetic linkage and physical map information of chickpea genome revealed correspondence of 1.8 cM SW QTL (*CaqSW1.1*) interval with 1,366,372 bp [CaSNP7 (5,380,861 bp) to CaSNP12 (1,904,458 bp)] genomic region on chromosome 1. The proportion of phenotypic variance explained (*R*^2^) by *CaqSW1.1* QTL was 47.6%. The identified *CaqSW1.1* QTL exhibiting consistent phenotypic expression, including major effect on SW trait variation across three geographical locations as well as years/seasons in field, was considered as ‘robust QTL’ as per Saxena *et al.*^24^ The positive additive gene effect of this QTL for increasing seed weight with effective allelic contribution from high seed weight genotype ICC 15061 was evident. In addition, one genomic region [CaSNP50 (52.6 cM) to CaSNP55 (60.2 cM)] underlying a robust SW QTL (*CaqSW1.2*) (8.7% *R*^2^ at LOD 5.4) covered with four SSR and SNP markers was identified and mapped on chromosome 1. This 7.6 cM target QTL interval showing positive additive effect corresponded to 139,393 bp [CaSNP50 (5,537,208 bp) to CaSNP55 (5,676,601 bp)] genomic region on chromosome 1.

### 3.4. Delineation of a candidate gene at the major seed weight QTL interval by integrating QTL-seq and classical QTL mapping with differential expression profiling

The correspondence of QTL-seq outcomes with classical QTL mapping inferred that one narrow 0.6 cM [3.9 (CaSNP8) to 4.5 (CaSNP10) cM] robust QTL (*CaqSW1.1*) interval harbouring a 35 kb major genomic region [836,859 (CaSNP8) to 872,247 (CaSNP10) kb] on chromosome 1 was associated with 100-seed weight in chickpea (Fig. 3C). The structural and functional annotation of this *CaqSW1.1* QTL region with *desi* genome annotation database identified six protein-coding candidate genes (Fig. 3D). Remarkably, one SNP locus (CaSNP8: G/A) in the coding region of constitutive photomorphogenic9 (COP9) signalosome complex subunit 8 (*CSN8*) gene showing tight linkage with a major SW robust QTL (*CaqSW1.1*) (based on single marker analysis of traditional QTL mapping) was considered as one of the potential candidate regulating seed weight in chickpea.

**Figure 4.**
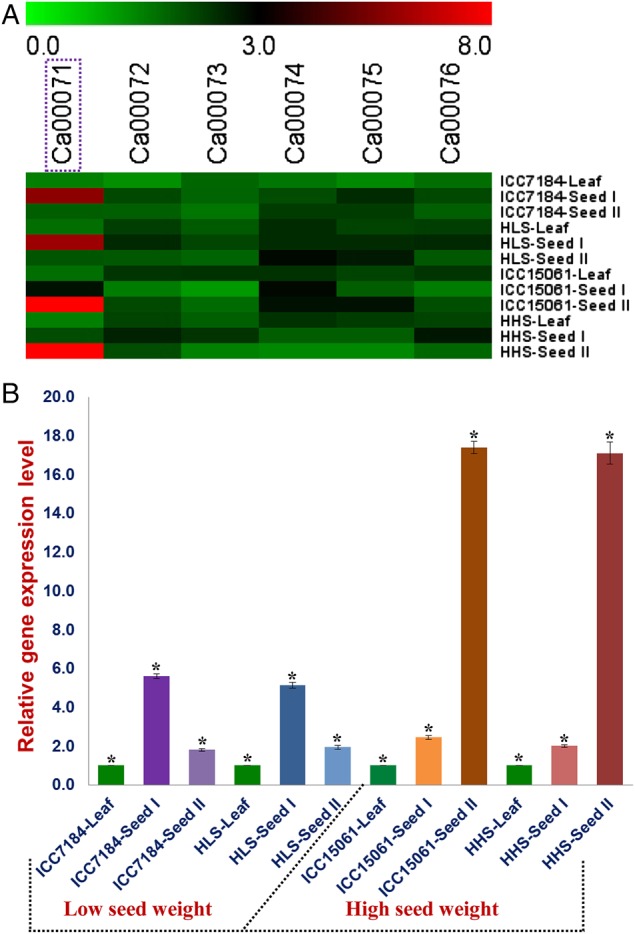
(A) Hierarchical cluster display represented differential expression profiles of six genes underlying a major SW QTL (*CaqSW1.1*) (identified by QTL-seq and traditional QTL mapping) in diverse vegetative (leaf) and reproductive (seed) tissues and two seed developmental stages of high and low seed weight parental genotypes and representative homozygous individuals of an intra-specific mapping population (ICC 7184 × ICC 15061). The average log signal expression values of genes in various tissues and developmental stages was denoted at the top with a colour scale; in which green, black and red color signify low, medium and high level of expression, respectively. One *CSN8 desi* gene (Ca00071) showing seed-specific expression, including pronounced differential up-/down-regulation in high and low seed weight mapping individuals and parental genotypes during seed development, is marked with violet box. The structural and functional annotations of six genes are mentioned in the Supplementary Table S2. The tissues/stages and genes used for expression profiling are indicated on the right and top side of expression map, respectively. An endogenous control *elongation factor-1 alpha* was used in quantitative RT–PCR assay for normalization of the expression values across different tissues/developmental stages of parents and mapping individuals. HLS: homozygous low seed weight; HHS: homozygous high seed weight; S1: seed development stage 1 (10–20 days after podding/DAP) and S2: seed development stage 2 (21-30 DAP). (B) Differential expression profiling of one coding SNP-carrying *CSN* gene harbouring a major SW QTL (*CaqSW1.1*) (identified by QTL-seq and traditional QTL mapping) in two seed developmental stages of high and low seed weight mapping individuals and parental genotypes (ICC 7184 and ICC 15061) compared with their respective vegetative leaf tissues using the quantitative RT–PCR assay. The gene expression in leaf tissues of mapping individuals and parents was considered as reference calibrator and assigned as 1. Each bar denotes the mean (± standard error) of three independent biological replicates with two technical replicates for each sample used in RT–PCR assay. *Significant differences (LSD-ANOVA significance test) in gene expression at two seed developmental stages of low and high seed weight mapping individuals and parents compared with leaf at *P* < 0.01. This figure is available in black and white in print and in colour at *DNA Research* online.

To delineate the potential candidate gene regulating seed weight in chickpea, differential expression profiling of six genes annotated at the 35 kb major genomic region underlying a *CaqSW1.1* QTL was performed. The RNA isolated from leaves and two seed developmental stages of low and high seed weight representative homozygous mapping individuals and parental genotypes was amplified with the six gene-based primers using semi-quantitative and quantitative RT–PCR assays (Fig. 4A). One SNP (G/A)-carrying *CSN8* gene of these, at *CaqSW1.1* QTL interval, exhibited seed-specific expression (compared with leaves) as well as pronounced up-regulation (>7-folds) in high seed weight parental genotype (ICC 15061) and homozygous mapping individuals during seed development (Fig. 4B). However, this gene was down-regulated (>3-folds) in seed developmental stages of low seed weight parental genotype (ICC 7184) and homozygous mapping individuals. These findings indicate that a *CSN*8 gene localized at the major SW QTL interval (*CaqSW1.1*) could be a potential candidate regulating seed weight in chickpea.

### 3.5. Mining of novel allelic variants for understanding the natural allelic diversity in a seed weight-regulating gene

**Figure 5.**
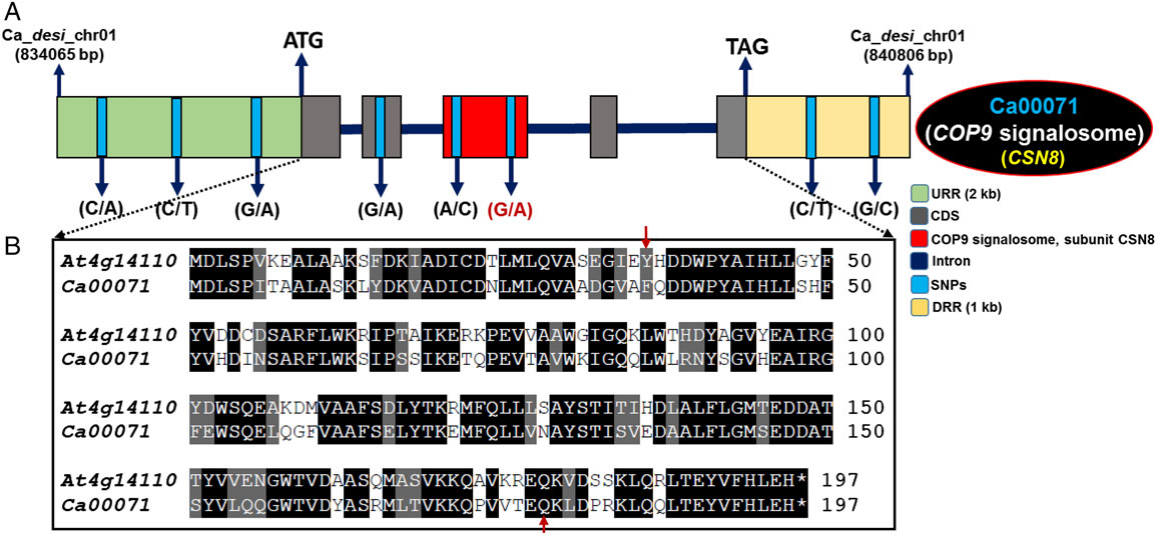
(A) Structural annotation of one candidate seed weight-regulating CSN8-domain containing COP9 signalosome gene delineated at *CaqSW1.1* major QTL interval by integrating QTL-seq with classical QTL mapping and differential expression profiling. Diverse coding (functional domain) and non-coding upstream (URR) and downstream (DRR) regulatory regions of gene are illustrated. One functionally relevant potential SNP (G/A) identified in the CDS of a *CSN* gene possibly regulating seed weight and development in chickpea is highlighted with red colour font. The cloned amplicon sequencing of this gene among 94 diverse wild and cultivated chickpea genotypes, including mapping parents and individuals constituting the LSB and HSB discovered altogether eight SNPs. This includes seven SNPs particularly mined from wild species/genotypes of primary, secondary and tertiary gene pools and one remaining coding SNP (G/A) from cultivated *desi* and *kabuli* genotypes, mapping parents and individuals of LSB and HSB. CDS: coding sequences. (B) Multiple sequence alignment depicting the amino acid sequence conservation of *desi* chickpea *CSN8* gene (Ca00071) with its orthologous gene (At4g14110) in *Arabidopsis thaliana* (*At*). The amino acid sequences with >50% identity and similarity between two gene orthologs are shaded with black and grey colour, respectively. The functional domain region of gene is marked with red arrows. This figure is available in black and white in print and in colour at *DNA Research* online.

To infer the natural allelic diversity (functional molecular diversity), one SW-governing *CSN*8 gene validated by QTL-seq, classical QTL mapping and expression profiling was selected. The cloned fragments amplified from entire coding and non-coding regions (including 2 kb upstream and 1 kb downstream regulatory regions) of this 6,741 bp gene were sequenced among 94 cultivated and wild chickpea genotypes (encompassing primary, secondary and tertiary gene pools) along with mapping parents and individuals constituting the LSB and HSB (Fig. 5A). This analysis identified eight SNPs of which seven SNPs (five upstream and downstream regulatory and two coding SNPs) were polymorphic exclusively among the genotypes belonging to wild species. The remaining one coding SNP (G/A) tightly linked to a major *CaqSW1.1* QTL exhibited differentiation specifically among the cultivated *desi* and *kabuli* genotypes. This coding SNP further discriminated low seed weight mapping parental genotypes and all individuals of LSB from the high seed weight mapping parents and HSB individuals. A higher nucleotide diversity of SNPs discovered from a SW-associated *CSN8* gene in the wild species/accessions (mean θπ: 0.31 and θω: 0.28) compared with cultivated species/accessions (0.12 and 0.10) was evident.

## 4. Discussion

The present study identified and mapped one major genomic region harbouring a robust 100-seed weight QTL (*CaqSW1.1*) on chromosome 1 using an intra-specific *desi* chickpea mapping population via whole-genome NGS-based high-throughput QTL-seq approach. This QTL-seq (based on SNP-index)-derived SW QTL (*CaqSW1.1*) was further validated by SNP and SSR marker-based traditional QTL mapping (at higher LOD >19). This suggests the validity and robustness of QTL-seq as a strategy for quick and efficient scanning of major QTL at a genome-wide scale in chickpea. The advantages of QTL-seq vis-a-vis other available traditional QTL mapping approaches (involving near-isogenic and recombinant inbred lines, Tuinstra *et al.*^61^; Monforte and Tanksley^62^; Loudet *et al.*^63^) to identify major QTLs governing seedling vigour, blast resistance and flowering time in crop plants (including rice and cucumber) for instance, have been recently reported.^40,41^ Moreover, our study ascertained the wider applicability of QTL-seq approach both in preliminary as well as much advanced generation mapping populations (RILs) by identifying a major seed weight-regulatory QTL in an intra-specific F_4_ mapping population of chickpea. The narrowing down of 1.8 cM *CaqSW1.1* major QTL interval [CaSNP7 (3.7 cM) to CaSNP12 (5.5 cM)] identified by classical QTL mapping into 0.6 cM seed weight-associated QTL region [CaSNP8 (3.9 cM) to -CaSNP10 (4.5 cM)] through QTL-seq was evident. This reflects the potential of QTL-seq over traditional QTL analysis for high-resolution genome mapping and subsequent fine mapping of target candidate genomic region harbouring a major trait-associated QTL. The underlying reason could be the NGS-based high-throughput genome-wide SNP scan between parental genotypes and individuals selected under study with contrasting phenotypes (low and high seed weight) of an intra-specific mapping population. A much closer examination of SNP-index and Δ (SNP-index) among parental genotypes as well as individuals constituting the HSB and LSB at major *CaqSW1.1* QTL indicated minor contribution of ∼20% low seed weight alleles in high seed weight parental genotypes and mapping individuals. This is in line with transgressive segregation and quantitative genetic inheritance pattern of 100-seed weight observed in the intra-specific mapping population under study. The detection of another minor (<10% *R*^2^ at LOD 5.4) SW QTL (*CaqSW1.2*) in chromosome 1 through traditional QTL mapping infers the possible shortcoming of QTL-seq approach to identify minor QTLs explaining low phenotypic variation specifically for quantitative agronomic traits. Further large-scale validation of these identified major (*CaqSW1.1*) and minor (*CaqSW1.2*) SW QTLs in diverse genetic background and/or through fine mapping/positional cloning is essential prior to their implementation in genomics-assisted breeding of chickpea for higher seed weight and yield.

The integration of QTL-seq with traditional QTL mapping (combining QTL-linked/flanking marker genetic and physical mapping information) narrowed down the 1.37 Mb [CaSNP7 (5,380,861 bp) to CaSNP12 (1,904,458 bp) containing 177 genes] major seed weight QTL region (*CaqSW1.1*) into a 35 kb physical interval [CaSNP8 (836,859 bp) to CaSNP10 (872,247 bp) carrying six genes] on chromosome 1, which explained ∼47.6% of the phenotypic variation of 100-seed weight. The differential expression profiling of six protein-coding genes annotated at this target genomic region further delimited the 35 kb physical interval into a coding SNP (G/A)-carrying potential *CSN8* gene. The seed-specific expression as well as pronounced differential up-/down-regulation of this candidate gene in high and low seed weight parental genotypes and homozygous mapping individuals during seed development was apparent. However, we could not find any SNPs in the 2 kb upstream and 1 kb downstream regulatory regions of a SW-associated *CSN* gene that showed differentiation among the cultivated *desi* and *kabuli* genotypes, including mapping parents and individuals of LSB and HSB. Therefore, speculating any possible correlation between regulatory sequence polymorphism and molecular mechanism underlying differential transcript accumulation of *CSN* gene during seed development especially in cultivated chickpea requires further experimentation. The sequence variant analysis targeting beyond the 2 kb upstream and 1 kb downstream regulatory regions of *CSN* gene may provide additional clues regarding its differential regulation during seed development in chickpea. The functional relevance of coding gene-derived SNPs for regulating diverse agronomic traits, including seed (grain) size/weight, have been well demonstrated in rice and chickpea.^64–67,20,21^ These findings collectively inferred that a combinatorial approach of QTL-seq, traditional QTL mapping and differential gene expression profiling have the potential to delineate one coding SNP (G/A)-carrying *CSN8* candidate gene at a major QTL interval regulating seed weight in chickpea. The implication of integrated genomic approach (combining genetic and association mapping with transcript profiling) to delineate potential candidate genes harbouring major trait-associated QTLs has been well documented in chickpea.^20,21,24,68^ The non-congruence of two SW QTLs (*CaqSW1.1* and *CaSW1.2*) identified in our study with that of previously reported known SW QTLs^2,7,20,21,24,25,68–71^ (comparing genetic/physical positions of these QTLs-linked/flanking markers mapped on diverse intra-/inter-specific linkage maps) was observed. This suggests the population-specific genetic inheritance pattern of our identified novel QTLs regulating seed weight in chickpea. Henceforth, our proposed integrated strategy can be employed for rapid identification of major potential genes, QTLs and alleles associated with qualitative as well as quantitative traits in diverse crop plants. The novel allelic variants mined from the diverse coding and non-coding sequence components of one seed weight-associated *CSN8* gene revealed a dramatic reduction of natural allelic diversity in cultivated species/genotypes in contrast to wild species/genotypes encompassing primary, secondary and tertiary gene pools. In spite of higher allelic diversity in wild species/genotypes, one seed weight-associated locus (G/A) in *CSN8* gene was completely absent from all the wild genotypes. However, this potential seed weight-regulating gene locus was present in all the *desi* and *kabuli* genotypes of cultivated species. This clearly reflects the possible effect of strong artificial and/or natural selection pressure on this seed weight gene locus during chickpea domestication. Further analysis involving all the natural allelic variants mined and potential locus targeted by natural and/or artificial selection in a SW-governing gene is required to gain a deeper insight into the complex seed weight trait evolution in chickpea. This will further assist us to decipher the molecular mechanisms underlying fixation of such complex quantitative trait in domesticated chickpea that are adapted to diverse agro-climatic conditions.

The COP9 signalosome complex subunit 8 (*CSN8*) chickpea gene homolog having 83% amino acid sequence conservation with *Arabidopsis thaliana* gene (At4g14110) (Fig. 5B) is known to be a major factor controlling growth and development in multiple plant species, including *Arabidopsis*.^72–76^ This evolutionary conserved multi-protein gene complex is said to be involved in regulation of various E3 ubiquitin ligases and auxin response-mediated developmental pathways in crop plants.^72,73^ However, functional validation and a detailed molecular characterization of this gene are required to understand its definite role in seed weight regulation in chickpea. Therefore, a major seed weight-regulating *CSN* gene identified at the robust QTL interval by integrating QTL-seq and classical QTL mapping with differential expression profiling, once functionally validated could be utilized as a potential candidate for marker-assisted genetic improvement of chickpea for enhancing its seed weight as well as yield.

## Supplementary data

Supplementary data are available at www.dnaresearch.oxfordjournals.org.

## Funding

The authors acknowledge the financial support for this research study provided by a research grant from the Department of Biotechnology (DBT), Government of India (102/IFD/SAN/2161/2013-14). S.D. and A.K. acknowledge the CSIR (Council of Scientific and Industrial Research) and DBT for Junior/Senior Research Fellowship awards. Funding to pay the Open Access publication charges for this article was provided by the National Institute of Plant Genome Research (NIPGR).

## Supplementary Material

Supplementary Data

## References

[1] Upadhyaya H.D. Kumar S. Gowda C.L.L. Singh S. 2006, Two major genes for seed size in chickpea (*Cicer arietinum L*.), Euphytica, 147, 311–15.

[2] Gowda C.L.L. Upadhyaya H.D. Dronavalli N. Singh S. 2011, Identification of large-seeded high-yielding stable kabuli chickpea germplasm lines for use in crop improvement, Crop Sci., 51, 198–209.

[3] Cho S. Kumar J. Shultz J.F. Anupama K. Tefera F. Muehlbauer F.J. 2002, Mapping genes for double podding and other morphological traits in chickpea, Euphytica, 125, 285–92.

[4] Rakshit S. Winter P. Tekeoglu M. 2003, DAF marker tightly linked to a major locus for *Ascochyta* blight resistance in chickpea (*Cicer arietinum* L.), Euphytica, 132, 23–30.

[5] Cobos M.J. Fernandez M. Rubio J. 2005, Linkage map of chickpea (*Cicer arietinum* L.) based on populations from *kabuli* × *desi* crosses: location of genes for resistance to *Fusarium* wilt race 0, Theor. Appl. Genet., 110, 1347–53. 15806343 10.1007/s00122-005-1980-1

[6] Cobos M. Rubio J. Strange R.N. Moreno M.T. Gil J. Millán T. 2006, A new QTL for *Ascochyta* blight resistance in an RIL population derived from an interspecific cross in chickpea, Euphytica, 149, 105–11.

[7] Cobos M.J. Winter P. Kharrat M. 2009, Genetic analysis of agronomic traits in a wide cross of chickpea, Field Crop Res., 111, 130–6.

[8] Lichtenzveig J. Bonfil D.J. Zhang H.B. Shtienberg D. Abbo S. 2006, Mapping quantitative trait loci in chickpea associated with time to flowering and resistance to *Didymella rabiei* the causal agent of *Ascochyta* blight, Theor. Appl. Genet., 113, 1357–69. 17016689 10.1007/s00122-006-0390-3

[9] Radhika P. Gowda S.J.M. Kadoo N.Y. 2007, Development of an integrated intraspecific map of chickpea (*Cicer arietinum* L.) using two recombinant inbred line populations, Theor. Appl. Genet., 115, 209–16. 17503013 10.1007/s00122-007-0556-7

[10] Tar'an B. Warkentin T.D. Tullu A. Vanderberg A. 2007, Genetic mapping of *Ascochyta* blight resistance in chickpea (*Cicer arietinum*) using a simple sequence repeat linkage map, Genome, 50, 26–34. 17546068 10.1139/g06-137

[11] Madrid E. Rubiales D. Moral A. 2008, Mechanism and molecular markers associated with rust resistance in a chickpea interspecific cross (*Cicer arietinum* × *Cicer reticulatum*), Eur. J. Plant. Pathol., 121, 43–53.

[12] Anbessa Y. Taran B. Warkentin T.D. Tullu A. Vandenberg A. 2009, Genetic analyses and conservation of QTL for *Ascochyta* blight resistance in chickpea (*Cicer arietinum* L.), Theor. Appl. Genet., 119, 757–65. 19517090 10.1007/s00122-009-1086-2

[13] Gowda S.J.M. Radhika P. Kadoo N.Y. Mhase L.B. Gupta V.S. 2009, Molecular mapping of wilt resistance genes in chickpea, Mol. Breed, 24, 177–83.

[14] Anuradha C. Gaur P.M. Pande S. 2011, Mapping QTL for resistance to *Botrytis* grey mould in chickpea, Euphytica, 182, 1–9.

[15] Aryamanesh N. Nelson M.N. Yan G. Clarke H.J. Siddique K.H.M. 2010, Mapping a major gene for growth habit and QTLs for *Ascochyta* blight resistance and flowering time in a population between chickpea and *Cicer reticulatum*, Euphytica, 173, 307–19.

[16] Gowda C.L.L. Upadhyaya H.D. Dronavalli N. Singh S. 2011, Identification of large-seeded high-yielding stable *kabuli* chickpea germplasm lines for use in crop improvement, Crop Sci., 5, 198–209.

[17] Kumar A. Choudhary A.K. Solanki R.K. Pratap A. 2011, Towards marker-assisted selection in pulses: a review, Plant Breed., 130, 297–313.

[18] Rehman A.U. Malhotra R.S. Bett K. Tar'an B. Bueckert R. Warkentin T.D. 2011, Mapping QTL associated with traits affecting grain yield in chickpea (*Cicer arietinum* L.) under terminal drought stress, Crop Sci., 51, 450–63.

[19] Vadez V. Krishnamurthy L. Thudi M. 2012, Assessment of ICCV2 × JG62 chickpea progenies shows sensitivity of reproduction to salt stress and reveals QTLs for seed yield and yield components, Mol. Breed., 30, 9–21.

[20] Kujur A. Bajaj D. Saxena M.S. 2013, Functionally relevant microsatellite markers from chickpea transcription factor genes for efficient genotyping applications and trait association mapping, DNA Res., 20, 355–74. 23633531 10.1093/dnares/dst015 PMC3738162

[21] Kujur A. Bajaj D. Saxena M.S. 2014, An efficient and cost-effective approach for genic microsatellite marker-based large-scale trait association mapping: identification of candidate genes for seed weight in chickpea, Mol. Breed., 34, 241–65.

[22] Sabbavarapu M.M. Sharma M. Chamarthi S.K. 2013, Molecular mapping of QTLs for resistance to *Fusarium* wilt (race 1) and *Ascochyta* blight in chickpea (*Cicer arietinum* L.), Euphytica, 193, 121–33.

[23] Ali L. Madrid E. Varshney R.K. 2014, Mapping and identification of a *Cicer arietinum NSP2* gene involved in nodulation pathway, Theor. Appl. Genet., 127, 481–8. 24247237 10.1007/s00122-013-2233-3

[24] Saxena M.S. Bajaj D. Das S. 2014, An integrated genomic approach for rapid delineation of candidate genes regulating agro-morphological traits in chickpea, DNA Res., 21, 695–710. 25335477 10.1093/dnares/dsu031 PMC4263302

[25] Stephens A. Lombardi M. Cogan N.O.I. 2014, Genetic marker discovery, intraspecific linkage map construction and quantitative trait locus analysis of *Ascochyta* blight resistance in chickpea (*Cicer arietinum* L.), Mol. Breed, 33, 297–313.

[26] Varshney R.K. Murali Mohan S. Gaur P.M. 2013, Achievements and prospects of genomics-assisted breeding in three legume crops of the semi-arid tropics, Biotechnol. Adv., 31, 1120–34. 23313999 10.1016/j.biotechadv.2013.01.001

[27] Varshney R.K. Thudi M. Nayak S.N. 2014, Genetic dissection of drought tolerance in chickpea (*Cicer arietinum* L.), Theor. Appl. Genet., 127, 445–62. 24326458 10.1007/s00122-013-2230-6 PMC3910274

[28] Varshney R.K. Gaur P.M. Chamarthi S.K. 2013, Fast-track introgression of “QTL-hotspot” for root traits and other drought tolerance trait in JG 11, an elite and leading variety of chickpea (*Cicer arietinum* L.), Plant Genome, 6, 1–26.

[29] Varshney R.K. Mohan S.M. Gaur P.M. 2014, Marker-assisted backcrossing to introgress resistance to *Fusarium* wilt race 1 and *Ascochyta* blight in C214, an elite cultivar of chickpea, Plant Genome, 7, 1–11.

[30] Hiremath P.J. Kumar A. Penmetsa R.V. 2012, Large-scale development of cost-effective SNP marker assays for diversity assessment and genetic mapping in chickpea and comparative mapping in legumes, Plant Biotechnol. J., 10, 716–32. 22703242 10.1111/j.1467-7652.2012.00710.x PMC3465799

[31] Gaur R. Azam S. Jeena G. 2012, High-throughput SNP discovery and genotyping for constructing a saturated linkage map of chickpea (*Cicer arietinum* L.), DNA Res., 19, 357–73. 22864163 10.1093/dnares/dss018 PMC3473369

[32] Roorkiwal M. Sawargaonkar S.L. Chitikineni A. 2013, Single nucleotide polymorphism genotyping for breeding and genetics applications in chickpea and pigeonpea using the BeadXpress platform, Plant Genome, 6, 1–10.

[33] Elshire R.J. Glaubitz J.C. Sun Q. 2011, A robust, simple genotyping-by-sequencing (GBS) approach for high diversity species, PLoS ONE, 6, e19379. 21573248 10.1371/journal.pone.0019379 PMC3087801

[34] Poland J.A. Brown P.J. Sorrells M.E. Jannink J.L. 2012, Development of high-density genetic maps for barley and wheat using a novel two-enzyme genotyping-by-sequencing approach, PLoS ONE, 7, e32253. 22389690 10.1371/journal.pone.0032253 PMC3289635

[35] Poland J.A. Rife T.W. 2012, Genotyping-by-sequencing for plant breeding and genetics, Plant Genome, 5, 92–102.

[36] Beissinger T.M. Hirsch C.N. Sekhon R.S. 2013, Marker density and read depth for genotyping populations using genotyping-by-sequencing, Genetics, 193, 1073–81. 23410831 10.1534/genetics.112.147710 PMC3606087

[37] Crossa J. Beyene Y. Kassa S. 2013, Genomic prediction in maize breeding populations with genotyping-by-sequencing, G3 (Bethesda), 3, 1903–26. 24022750 10.1534/g3.113.008227 PMC3815055

[38] Sonah H. Bastien M. Iquira E. 2013, An improved genotyping by sequencing (GBS) approach offering increased versatility and efficiency of SNP discovery and genotyping, PLoS ONE, 8, e54603. 23372741 10.1371/journal.pone.0054603 PMC3553054

[39] Spindel J. Wright M. Chen C. 2013, Bridging the genotyping gap: using genotyping by sequencing (GBS) to add high-density SNP markers and new value to traditional bi-parental mapping and breeding populations, Theor. Appl. Genet., 126, 2699–716. 23918062 10.1007/s00122-013-2166-x

[40] Jaganathan D. Thudi M. Kale S. 2014, Genotyping-by-sequencing based intra-specific genetic map refines a “QTL-hotspot” region for drought tolerance in chickpea, Mol. Genet. Genomics, 290, 559–71. 25344290 10.1007/s00438-014-0932-3 PMC4361754

[41] Liu H. Bayer M. Druka A. 2014, An evaluation of genotyping by sequencing (GBS) to map the Breviaristatum-e (ari-e) locus in cultivated barley, BMC Genomics, 15, 104. 24498911 10.1186/1471-2164-15-104 PMC3922333

[42] Takagi H. Abe A. Yoshida K. 2013, QTL-seq: rapid mapping of quantitative trait loci in rice by whole genome resequencing of DNA from two bulked populations, Plant J., 74, 174–83. 23289725 10.1111/tpj.12105

[43] Lu H. Lin T. Klein J. 2014, QTL-seq identifies an early flowering QTL located near flowering locus *T* in cucumber, Theor. Appl. Genet., 127, 1491–9. 24845123 10.1007/s00122-014-2313-z

[44] Jain M. Misra G. Patel R.K. 2013, A draft genome sequence of the pulse crop chickpea (*Cicer arietinum* L.), Plant J., 74, 715–29. 23489434 10.1111/tpj.12173

[45] Langmead B. Salzberg S.L. 2012, Fast gapped-read alignment with Bowtie 2, Nat. Methods, 9, 357–9. 22388286 10.1038/nmeth.1923 PMC3322381

[46] Li H. Durbin R. 2009, Fast and accurate short read alignment with Burrows-Wheeler transform, Bioinformatics, 25, 1754–60. 19451168 10.1093/bioinformatics/btp324 PMC2705234

[47] Abe A. Kosugi S. Yoshida K. 2012, Genome sequencing reveals agronomically important loci in rice using MutMap, Nat. Biotechnol., 30, 174–8. 22267009 10.1038/nbt.2095

[48] Parida S.K. Mukerji M. Singh A.K. Singh N.K. Mohapatra T. 2012, SNPs in stress-responsive rice genes: validation, genotyping, functional relevance and population structure, BMC Genomics, 13, 426. 22921105 10.1186/1471-2164-13-426 PMC3562522

[49] Winter P. Pfaff T. Udupa S.M. 1999, Characterization and mapping of sequence-tagged microsatellite sites in the chickpea (*Cicer arietinum* L.) genome, Mol. Gen. Genet., 262, 90–101. 10503540 10.1007/s004380051063

[50] Winter P. Benko-Iseppon A.M. Hüttel B. 2000, A linkage map of chickpea (*Cicer arietinum* L.) genome based on recombinant inbred lines from a *C. arietinum* × *C. reticulatum* cross: localization of resistance genes for *Fusarium* wilt races 4 and 5, Theor. Appl. Genet., 101, 1155–63.

[51] Thudi M. Li Y. Jackson S.A. May G.D. Varshney R.K. 2012, Current state-of-art of sequencing technologies for plant genomics research, Brief Funct. Genomics, 11, 3–11. 22345601 10.1093/bfgp/elr045

[52] Jhanwar S. Priya P. Garg R. Parida S.K. Tyagi A.K. Jain M. 2012, Transcriptome sequencing of wild chickpea as a rich resource for marker development, Plant Biotechnol. J., 10, 690–702. 22672127 10.1111/j.1467-7652.2012.00712.x

[53] Nayak S.N. Zhu H. Varghese N. 2010, Integration of novel SSR and gene-based SNP marker loci in the chickpea genetic map and establishment of new anchor points with *Medicago truncatula* genome, Theor. Appl. Genet., 120, 1415–41. 20098978 10.1007/s00122-010-1265-1 PMC2854349

[54] Gaur R. Sethy N.K. Choudhary S. Shokeen B. Gupta V. Bhatia S. 2011, Advancing the STMS genomic resources for defining new locations on the intraspecific genetic linkage map of chickpea (*Cicer arietinum* L.), BMC Genomics, 12, 117. 21329497 10.1186/1471-2164-12-117 PMC3050819

[55] Thudi M. Bohra A. Nayak S.N. 2011, Novel SSR markers from BAC-end sequences, DArT arrays and a comprehensive genetic map with 1,291 marker loci for chickpea (*Cicer arietinum* L.), PLoS ONE, 6, e27275. 22102885 10.1371/journal.pone.0027275 PMC3216927

[56] Choudhary P. Khanna S.M. Jain P.K. 2012, Genetic structure and diversity analysis of the primary gene pool of chickpea using SSR markers, Genet. Mol. Res., 11, 891–905. 22576917 10.4238/2012.April.10.5

[57] Wang W. Wang L. Chen C. 2011, *Arabidopsis CSLD1* and *CSLD4* are required for cellulose deposition and normal growth of pollen tubes, J. Exp. Bot., 62, 5161–77. 21765162 10.1093/jxb/err221 PMC3193019

[58] Van Ooijen J.W. 2009, MapQTL 6: software for the mapping of quantitative trait loci in experimental populations of diploid species, Kyazma BV: Wageningen, Netherlands.

[59] Saxena M.S. Bajaj D. Kujur A. 2014, Natural allelic diversity, genetic structure and linkage disequilibrium pattern in wild chickpea, PLoS ONE, 9, e107484. 25222488 10.1371/journal.pone.0107484 PMC4164632

[60] Tamura K. Peterson D. Peterson N. Stecher G. Nei M. Kumar S. 2011, MEGA5: molecular evolutionary genetics analysis using maximum likelihood, evolutionary distance, and maximum parsimony methods, Mol. Biol. Evol., 28, 2731–9. 21546353 10.1093/molbev/msr121 PMC3203626

[61] Tuinstra M.R. Ejeta G. Goldsbrough P.B. 1997, Heterogeneous inbred family (HIF) analysis: a method for developing near-isogenic lines that differ at quantitative trait loci, Theor. Appl. Genet., 95, 1005–11.

[62] Monforte A.J. Tanksley S.D. 2000, Development of a set of near isogenic and backcross recombinant inbred lines containing most of the *Lycopersicon hirsutum* genome in a *L. esculentum* genetic background: a tool for gene mapping and gene discovery, Genome, 43, 803–13. 11081970

[63] Loudet O. Gaudon V. Trubuil A. Daniel-Vedele F. 2005, Quantitative trait loci controlling root growth and architecture in *Arabidopsis thaliana* confirmed by heterogeneous inbred family, Theor. Appl. Genet., 110, 742–53. 15678326 10.1007/s00122-004-1900-9

[64] Fan C. Xing Y. Mao H. 2006, *GS3*, a major QTL for grain length and weight and minor QTL for grain width and thickness in rice, encodes a putative transmembrane protein, Theor. Appl. Genet., 112, 1164–71. 16453132 10.1007/s00122-006-0218-1

[65] Mao H. Sun S. Yao J. 2010, Linking differential domain functions of the GS3 protein to natural variation of grain size in rice, Proc. Natl Acad. Sci. USA, 107, 19579–84. 20974950 10.1073/pnas.1014419107 PMC2984220

[66] Li Y. Fan C. Xing Y. 2011, Natural variation in *GS5* plays an important role in regulating grain size and yield in rice, Nat. Genet., 43, 1266–70. 22019783 10.1038/ng.977

[67] Zhang X. Wang J. Huang J. 2012, Rare allele of *OsPPKL1* associated with grain length causes extra-large grain and a significant yield increase in rice, Proc. Natl Acad. Sci. USA, 109, 21534–39. 23236132 10.1073/pnas.1219776110 PMC3535600

[68] Bajaj D. Saxena M.S. Kujur A. 2015, Genome-wide conserved non-coding microsatellite (CNMS) marker-based integrative genetical genomics for quantitative dissection of seed weight in chickpea, J. Exp. Bot., 66, 1271–90. 25504138 10.1093/jxb/eru478 PMC4339591

[69] Cobos M.J. Rubio J. Ferna'ndez-Romero M.D. 2007, Population derived from a *kabuli* x *desi* cross, Ann. Appl. Biol., 151, 33–42.

[70] Hossain S. Ford R. McNeil D. Pittock C. Panozzo J.F. 2010, Inheritance of seed size in chickpea (*Cicer arietinum L*.) and identification of QTL based on 100-seed weight and seed size index, Aust. J. Crop Sci., 4, 126–35.

[71] Thudi M. Upadhyaya H.D. Rathore A. 2014, Genetic dissection of drought and heat tolerance in chickpea through genome-wide and candidate gene-based association mapping approaches, PloS ONE, 9, e96758. 24801366 10.1371/journal.pone.0096758 PMC4011848

[72] Serino G. Deng X.W. 2003, The COP9 signalosome: regulating plant development through the control of proteolysis, Annu. Rev. Plant. Biol., 54, 165–82. 14502989 10.1146/annurev.arplant.54.031902.134847

[73] Schwechheimer C. Isono E. 2010, The COP9 signalosome and its role in plant development, Eur. J. Cell Biol., 89, 157–62. 20036030 10.1016/j.ejcb.2009.11.021

[74] Nakasone A. Fujiwara M. Fukao Y. 2012, SMALL ACIDIC PROTEIN1 acts with RUB modification components, the COP9 signalosome, and AXR1 to regulate growth and development of *Arabidopsis*, Plant Physiol., 160, 93–105. 22576848 10.1104/pp.111.188409 PMC3440233

[75] Esteve-Bruna D. Pérez-Pérez J.M. Ponce M.R. Micol J.L. 2013, *incurvata13*, a novel allele of AUXIN RESISTANT6, reveals a specific role for auxin and the SCF complex in *Arabidopsis* embryogenesis, vascular specification, and leaf flatness, Plant Physiol., 161, 1303–20. 23319550 10.1104/pp.112.207779 PMC3585598

[76] Franciosini A. Lombardi B. Iafrate S. 2013, The *Arabidopsis* COP9 SIGNALOSOME INTERACTING F-BOX KELCH 1 protein forms an SCF ubiquitin ligase and regulates hypocotyl elongation, Mol. Plant, 6, 1616–29. 23475998 10.1093/mp/sst045

