# Supplementary material for: Deploying QTL-seq for rapid delineation of a potential candidate gene underlying major trait-associated QTL in chickpea
Source: DNA Res. 2015 Apr 27;22(3):193–203. doi: 10.1093/dnares/dsv004 (PMC4463844; doi:10.1093/dnares/dsv004)
Supplement: Supplementary Data [file supp_22_3_193__index.html]

Deploying QTL-seq for rapid delineation of a potential candidate gene underlying major trait-associated QTL in chickpea — Supplementary Data 

# Deploying QTL-seq for rapid delineation of a potential candidate gene underlying major trait-associated QTL in chickpea

## Supplementary Data

Supplementary Data

**Files in this Data Supplement:**

- Supplementary Table 1 - pdf file
- Supplementary Table 2 - pdf file
