# Supplementary material for: Deploying QTL-seq for rapid delineation of a potential candidate gene underlying major trait-associated QTL in chickpea
Source: DNA Res. 2015 Apr 27;22(3):193–203. doi: 10.1093/dnares/dsv004 (PMC4463844; doi:10.1093/dnares/dsv004)
Supplement: Supplementary Data [file supp_dsv004_dsv004supp_table1.pdf]

Table S1: SNPs genetically mapped on chromosome 1 used for seed weight targeted traditional QTL mapping in chickpea

SNP IDs	Desi chromosomes	Physical positions (bp)	SNPs/SSRs	Upstream 200-bp flanking sequences/Forward primers (5'-3')	Downstream 200-bp flanking sequences/Reverse primers (5'-3')	Genetic positions (cM)
CaSNP1	*Ca-desi* -chr01	382862	(T/C)	TTATGGTTAACATCACTGTTCTATTTTTTTAATTATCTTTCAGGAAAGGTTGGAAAGAAGACGGCAGATGCCATATCACATGCATATAAATCTTGAGCTCCTGGAATCAGTGCATCTTATATCTGCTATGCTGCTGGAAGTTCCTAATATGGCAGCCAATGTTCATGATGCAAAGCGTAAGATCATTAGTAAGAATTTCCG	CGCTTGCTGGAGATCAGTGAGAAACAAACATTCACTGGTCCTCCTGAAAATGTTCGGGATCATGTTATGGCTGCCACGAGGGCTCTTAGTAAAGGAGACTTCAACAAGGCTTTTGACATTATTGCATCTCTTGATGTATGGAAATTTGTGAAAAATCGAGATGCCGTGCTAGAAATGCTGAAGGACAAAATCAAGGAGGAG	0
CaSNP2	*Ca-desi* -chr01	382898	(T/C)	CTTTCAGGAAAGGTTGGAAAGAAGACGGCAGATGCCATATCACATGCATATAAATCTTGAGCTCCTGGAATCAGTGCATCTTATATCTGCTATGCTGCTGGAAGTTCCTAATATGGCAGCCAATGTTCATGATGCAAAGCGTAAGATCATTAGTAAGAATTTCCGTCGCTTGCTGGAGATCAGTGAGAAACAAACATTCAC	GGTCCTCCTGAAAATGTTCGGGATCATGTTATGGCTGCCACGAGGGCTCTTAGTAAAGGAGACTTCAACAAGGCTTTTGACATTATTGCATCTCTTGATGTATGGAAATTTGTGAAAAATCGAGATGCCGTGCTAGAAATGCTGAAGGACAAAATCAAGGAGGAGGCACTTAGGACATACCTATTTACCTTCTCTTCATCG	1.1
CaSNP3	*Ca-desi* -chr01	527099	(A/G)	AATAAGAAATTTGGGTTATCATTAGCATTCCTTTGTAACTTCTTCATTCTCTTTTCACTGTCAATTTCCTACTTGTTAAAGTGCTGACACTGAAAGTTTCTATGATTTGTTTCTCATTCTTGCTGTATGCTCTAAATTTGCAGGCTCGGTATTTCTTCCAGCAGCTCATCTCAGGAGTTAGCTACTGCCATTGCATGGTAA	TTTAGTTTTTTTtGTTTTTTTtCAATGTGTTGGTGGTTTTTtGCCTGTCTTTTGGGGTTATTTTtCTATGTAATTTTtCTTCTTTTGCAGCAAATCTGCCATCGAGACTTGAAGCTCGAGAACACCTTATTGGATGGGAACCCTGCCCCGCGGCTCAAAATTTGCGATTTTGGTTATTCTAAGGTTCAGTTATGGAGTAAT	1.4
CaSSR1	*Ca-desi* -chr01		(TA113)	TCTGCAAAAACTATTACGTTAATACCA	TTGTGTGTAATGGATTGAGTATCTCTT	2.2
CaSNP4	*Ca-desi* -chr01	527113	(G/T)	GTTATCATTAGCATTCCTTTGTAACTTCTTCATTCTCTTTTCACTGTCAATTTCCTACTTGTTAAAGTGCTGACACTGAAAGTTTCTATGATTTGTTTCTCATTCTTGCTGTATGCTCTAAATTTGCAGGCTCGGTATTTCTTCCAGCAGCTCATCTCAGGAGTTAGCTACTGCCATTGCATGGTAAATTTAGTTTTTTTt	TTTTTTTtCAATGTGTTGGTGGTTTTTtGCCTGTCTTTTGGGGTTATTTTtCTATGTAATTTTtCTTCTTTTGCAGCAAATCTGCCATCGAGACTTGAAGCTCGAGAACACCTTATTGGATGGGAACCCTGCCCCGCGGCTCAAAATTTGCGATTTTGGTTATTCTAAGGTTCAGTTATGGAGTAATTTCAATGCCAAATG	2.6
CaSNP5	*Ca-desi* -chr01	527176	(T/C)	AAAGTGCTGACACTGAAAGTTTCTATGATTTGTTTCTCATTCTTGCTGTATGCTCTAAATTTGCAGGCTCGGTATTTCTTCCAGCAGCTCATCTCAGGAGTTAGCTACTGCCATTGCATGGTAAATTTAGTTTTTTTtGTTTTTTTtCAATGTGTTGGTGGTTTTTtGCCTGTCTTTTGGGGTTATTTTtCTATGTAATTT	tCTTCTTTTGCAGCAAATCTGCCATCGAGACTTGAAGCTCGAGAACACCTTATTGGATGGGAACCCTGCCCCGCGGCTCAAAATTTGCGATTTTGGTTATTCTAAGGTTCAGTTATGGAGTAATTTCAATGCCAAATGATTTGAATAAATTATTGAAGTCATACTTGACGTGTTTTtCTTCTGACAACATTTTGTTTTtCT	2.8
CaSNP6	*Ca-desi* -chr01	527181	(C/A)	GCTGACACTGAAAGTTTCTATGATTTGTTTCTCATTCTTGCTGTATGCTCTAAATTTGCAGGCTCGGTATTTCTTCCAGCAGCTCATCTCAGGAGTTAGCTACTGCCATTGCATGGTAAATTTAGTTTTTTTtGTTTTTTTtCAATGTGTTGGTGGTTTTTtGCCTGTCTTTTGGGGTTATTTTtCTATGTAATTTTtCTT	TTTTGCAGCAAATCTGCCATCGAGACTTGAAGCTCGAGAACACCTTATTGGATGGGAACCCTGCCCCGCGGCTCAAAATTTGCGATTTTGGTTATTCTAAGGTTCAGTTATGGAGTAATTTCAATGCCAAATGATTTGAATAAATTATTGAAGTCATACTTGACGTGTTTTtCTTCTGACAACATTTTGTTTTtCTAGTCA	3.3

SNP IDs	Desi chromosomes	Physical positions (bp)	SNPs/SSRs	Upstream 200-bp flanking sequences/Forward primers (5'-3')	Downstream 200-bp flanking sequences/Reverse primers (5'-3')	Genetic positions (cM)
CaSNP7	*Ca-desi* -chr01	538086	(G/T)	CAGCACAAGGTCTTCAAATTCCTCAGCTCAATTGGTT<br>GCAGCATGCTAATGCTCACCACAATTTCTCGTCCCAG<br>GCCAAGTTTTTGACCTCAAATTTCCTTTTCTCTATTCCA<br>ACTCACAAACCTTCCTCCAACGATGGGAGTGGCAGG<br>TATGTTATGCCTTTTAGTCTTTTAAAACAACAATTTTTT<br>tCTCTCCCTTTTT	TTTTGGTATGCAGCAATCTTTAGTGCTATTTGTCTTATG<br>ACATTGTATAGTGGGTAGTTTTTTGTcACTAGAGAAAA<br>GTTCTTAGACAATGCCATATCCTGATTTTGTAGCCTTTT<br>GCATATCATTACCAGCCTATTTTAGATATTATACTAGAA<br>CCCTATTGATAGAGATATATAGTATATAACATAACAAG<br>GATATAAG	3.7
CaSNP8	*Ca-desi* -chr01	836859	(G/A)	GGTTGGTTATATTATTGTTATGTTTTGCAATATTTTTA<br>TATTTTAGGGTTTTAATGTTGTGTTGTGATATTGCAGC<br>AACAGTGCTCGCTTTCTATGGAAATCGATTCCTTCTTC<br>AATCAAAGAAACCCAACCCGAAGTCACTGCTGTGTG<br>GAAAATTGGTCAACAGCTTTGGTTACGGAACTATTCC<br>GGAGTACATGAGGC	ATCCGTGGGTTTGAATGGAGTCAGGAACTCCAGGGATT<br>TGTTGCTGCTTTCTCAGGCAAAGCATTTAAGATTTTCTG<br>TTTTGTCTATTCAGCAGAGCTGCGATTGTTCTGTACAAA<br>CTCTTTGATCTTCACTATATTTAATGCATGATTCTATTCA<br>CTTTATCTTCAATTTGAATATAATTTTAAAGCTCTAACTG<br>GATGA	3.9
CaSSR2	*Ca-desi* -chr01		(TR43)	AGGACGAAACTATTCAAGGTAAGTAGA	AATTGAGATGGTATTAAATGGATAACG	4.1
CaSNP10	*Ca-desi* -chr01	872247	(G/A)	AAGAGAAACATACCACAACAACAGGTTTTGATTAGTT<br>CACCTTAAAAATCTCTCTTCTTCATGATTAACCTATTC<br>ACAAGCCAACTTGGTGTCAAAGCTGCTCAAGCATATT<br>GCAAAAGCTTGGAATGCAGATAAAGGATATCGATAA<br>TCCATGGTGAACATATCTTTCCCAACTTTGCCAAATTG<br>CAGAATAATCTTATC	TGTTCCGGTGGAGCTGGTTGAGATGGTGTTGGCGCGC<br>CAGCCGGTGGTTGAGTTGCAGCAATCAGCTGAAAGTTT<br>TTAACAGATGCAACTGTTACTCGTCCCCGGAAATTAAG<br>ACACCAGCATTGTAACTGTTCATGCCATCTAGGAGGTTT<br>GTTTTtCAGAACCAAAGGTCTCGTCTTGCCATCATCGTC<br>GTCATTAATA	4.5
CaSNP11	*Ca-desi* -chr01	872280	(C/T)	AGTTCACCTTAAAAATCTCTCTTCTTCATGATTAACCT<br>ATTCACAAGCCAACTTGGTGTCAAAGCTGCTCAAGCA<br>TATTGCAAAAGCTTGGAATGCAGATAAAGGATATCG<br>ATAATCCATGGTGAACATATCTTTCCCAACTTTGCCAA<br>ATTGCAGAATAATCTTATCGTGTTCCGGTGGAGCTGG<br>TTGAGATGGTGTTGG	GCGCCAGCCGGTGGTTGAGTTGCAGCAATCAGCTGAA<br>AGTTTTTAACAGATGCAACTGTTACTCGTCCCCGGAAAT<br>TAAGACACCAGCATTGTAACTGTTCATGCCATCTAGGA<br>GGTTTGTTTTtCAGAACCAAAGGTCTCGTCTTGCCATCA<br>TCGTCGTCATTAATAATGGAACCCCCGATTTCAGAAAA<br>CCGTGAACTG	4.7
CaSSR3	*Ca-desi* -chr01		(TA8)	AAAATTTGCACCCACAAAATATG	CTGAAAA (TAA)44TTATGGCAGGGAAAC	5
CaSNP12	*Ca-desi* -chr01	1904458	(G/A)	GCTCTTGTTTTGTGTTAGTGTACTTAGCAATTGCATTA<br>TCTTGGTTGTGCTGATAAGTGATACTCAAGTTGGTGT<br>TTTTCATGACAATTTTCAAGCAGATAAATGCGTATTG<br>ATATAGTGAGTGTGAATCTGAGCTTAGCTGCACCTAA<br>ATCATGTTTGACAAACTCAAGACAATGAGGGAAGGA<br>GAATCATCTAAGATAA	ATTTATATATATGTCTTTGAATTATTATCACTTCATCAAG<br>AACCAGATGCTGCAACCCGTTCTCGATTCTCTCTTGTAT<br>TATACGGAAGGAAGACCCATGTTATTATTTTAAGAAGA<br>TTATATTCTTTGTATGCAACTTGAAATTGAACAGCGTGC<br>CAATTTTGATTCGACTCTTTCCTAATTAATTATATGGTGA<br>GATAT	5.5

**SNP IDs**	**Desi chromosomes**	**Physical positions (bp)**	**SNPs/SSRs**	**Upstream 200-bp flanking sequences/Forward primers (5'-3')**	**Downstream 200-bp flanking sequences/Reverse primers (5'-3')**	**Genetic positions (cM)**
CaSNP13	*Ca-desi* -chr01	1904473	(C/T)	TAGTGTACTTAGCAATTGCATTATCTTGGTTGTGCTGATAAGTGATACTCAAGTTGGTGTTTTTCATGACAATTTTCAAGCAGATAAATGCGTATTGATATAGTGAGTGTGAATCTGAGCTTAGCTGCACCTAAATCATGTTTGACAAACTCAAGACAATGAGGGAAGGAGAATCATCTAAGATAAGATTTATATATATGT	TTTGAATTATTATCACTTCATCAAGAACCAGATGCTGCAACCCGTTCTCGATTCTCTCTTGTATTATACGGAAGGAAGACCCATGTTATTATTTTAAGAAGATTATATTCTTTGTATGCAACTTGAAATTGAACAGCGTGCCAATTTTGATTCGACTCTTTCCTAATTAATTATATGGTGAGATATGAGAGTTGAGGAATC	5.9
CaSNP14	*Ca-desi* -chr01	1906298	(A/C)	AACACTCGCAGATACCAAATTTGTTTCTATAATCCTAAATCGAGTCTCTTACGAAAATCTAGTTAATCTTGCACAAACAGGACTTTTGAAGATCATGTTAGATGCATTTATATCGGGATAAAAATAATGTTTGCATGAAAGGTTAGCTCCTACTATAGGAAATTGGAGACCCTTTTCAGCTGCAGTATGGTGCTGTAGCTA	ATAGGATCCACACTTTTCCCTCTGCACCCCcTACATCCTCTCTATCTCTTTCTTAATTTtCTTCGCTGATTGTCTCTAAATTTTTCATCATCTACTCTCTTCTCATTGTCTTATTtGCGCTATAGATGATGCACAGCAGCAGAGGATTCAAGCTCATGGAAGCCTACCTGCTCAATTCTACGGTGAAAAATGTGTCTCAAG	6.2
CaSNP15	*Ca-desi* -chr01	1906300	(T/G)	CACTCGCAGATACCAAATTTGTTTCTATAATCCTAAATCGAGTCTCTTACGAAAATCTAGTTAATCTTGCACAAACAGGACTTTTGAAGATCATGTTAGATGCATTTATATCGGGATAAAAATAATGTTTGCATGAAAGGTTAGCTCCTACTATAGGAAATTGGAGACCCTTTTCAGCTGCAGTATGGTGCTGTAGCTAAA	AGGATCCACACTTTTCCCTCTGCACCCCcTACATCCTCTCTATCTCTTTCTTAATTTtCTTCGCTGATTGTCTCTAAATTTTTCATCATCTACTCTCTTCTCATTGTCTTATTtGCGCTATAGATGATGCACAGCAGCAGAGGATTCAAGCTCATGGAAGCCTACCTGCTCAATTCTACGGTGAAAAATGTGTCTCAAGAA	6.5
CaSNP16	*Ca-desi* -chr01	1906308	(A/G)	GATACCAAATTTGTTTCTATAATCCTAAATCGAGTCTCTTACGAAAATCTAGTTAATCTTGCACAAACAGGACTTTTGAAGATCATGTTAGATGCATTTATATCGGGATAAAAATAATGTTTGCATGAAAGGTTAGCTCCTACTATAGGAAATTGGAGACCCTTTTCAGCTGCAGTATGGTGCTGTAGCTAAATAGGATCC	CACTTTTCCCTCTGCACCCCcTACATCCTCTCTATCTCTTTCTTAATTTtCTTCGCTGATTGTCTCTAAATTTTTCATCATCTACTCTCTTCTCATTGTCTTATTtGCGCTATAGATGATGCACAGCAGCAGAGGATTCAAGCTCATGGAAGCCTACCTGCTCAATTCTACGGTGAAAAATGTGTCTCAAGAACCCGTTAA	6.8
CaSNP17	*Ca-desi* -chr01	1906363	(G/A)	ATCTTGCACAAACAGGACTTTTGAAGATCATGTTAGATGCATTTATATCGGGATAAAAATAATGTTTGCATGAAAGGTTAGCTCCTACTATAGGAAATTGGAGACCCTTTTCAGCTGCAGTATGGTGCTGTAGCTAAATAGGATCCACACTTTTCCCTCTGCACCCCcTACATCCTCTCTATCTCTTTCTTAATTTtCTTC	CTGATTGTCTCTAAATTTTTCATCATCTACTCTCTTCTCATTGTCTTATTtGCGCTATAGATGATGCACAGCAGCAGAGGATTCAAGCTCATGGAAGCCTACCTGCTCAATTCTACGGTGAAAAATGTGTCTCAAGAACCCGTTAAAGTGTTTGATTCGGTGAATTTTCGCAATTTCCTCGTGCAATTTTTCtAGCAAACC	7.5

SNP IDs	Desi chromosomes	Physical positions (bp)	SNPs/SSRs	Upstream 200-bp flanking sequences/Forward primers (5'-3')	Downstream 200-bp flanking sequences/Reverse primers (5'-3')	Genetic positions (cM)
CaSNP18	*Ca-desi* -chr01	2067290	(C/A)	TTTCGAGGAGTCAGGAAGAGGCCATGGGGAAGATACGCCGCCGAGATTAGAGATCCGGGAAAGAAGAGCCGCGTTTGGCTCGGCACTTTCGACACGGCGGAAGAGGCTGCACGAGCCTACGACACCGCCGCACGTGATTTCCGTGGTTCTAAGGCTAAAACTAACTTCCCTTACCCTTCCGAAAATATAAAGATCCAAAGT	CTAGCCAAAGCAGCACCGTTGAATCCTCCAGCCGCGACCGTGACGCCGCCGCCGATGAATCTTATCCGCTTGACCTTAACTTATCCCCCGCTATCAACGGCTCCGTTCAGTTTCCTTTTCAGCATCGTTTCCCGTCGATGCCGCCAGCTGACAAGTTTTTTtATtATGACACCGTCGTACGCGCCGGCATGGTGAATTCCC	8.4
CaSNP19	*Ca-desi* -chr01	2084506	(G/T)	TCCTAAATAAGACGCAGTTGAGGTGTGCTCAGCCACCTGAACTCCACCGGAGACAGAAAAAGCACCGTCATGACGCCTAAGTCCAGCGACCGCAGCAACAGGTGGAGGCTGAGGAGTGTTAAAATCCTTGAGAACTCCAGTCTGAAGAAACTCAGTCACGACGAAGCTGCGCGAAGGGACAGGCGGCCATCGGTGATCAGA	GAAGGAGCGTTGAGATCAAGACAAAGACCAGCGGCAACCGGCGCCGGAGGAAAGTtAGTTTtAGCCTTAGCTCCACGGAGGGAACGAGCGGCGCCGTCGTAAGCAAGAGCAGCTTCCTCGGGAGTATCAAATGTTCCCAACCAAACACGTGTCTTCTTCCAAGGATCGCGAATCTCAGCTGCATAGCGTCCCCATGGTCGC	9.6
CaSNP20	*Ca-desi* -chr01	2213694	(C/T)	CTAATTTCCTGTGAATGTAGGTCCTCTGAAGTTAGTATCTCGCTGGAGTCGCCATTCCTTCTCCTTCAAACACTCCAGCAGAGACTGCGGCTCGTTTCTCAGTTCAGTCACAGCACAGCAGCCAAAAGAGCCTCCTGGTCTTTGACAATTGTTGTAAAGACTAGCTTTGTGTTCACCTGCATTGAGAAAACCTGGCTGCAA	GGCGGCGGACCTGGGGAGTCAATATATGTGTACTGGTTATTATACAACAAAGGATTGCCACGTCTGTTGAATTGATCAAACCTGTAAGTATTATCGTGATAGAGGAAATTTTCATGATTTTCAGGACTCGGAGTATTCATATAAGTCGGATGCATATAATTGTTGGCCCTCTCGGGTTTGTTGTTTTGTCTGTACCAATGC	9.8
CaSNP21	*Ca-desi* -chr01	2213700	(C/T)	TCCTGTGAATGTAGGTCCTCTGAAGTTAGTATCTCGCTGGAGTCGCCATTCCTTCTCCTTCAAACACTCCAGCAGAGACTGCGGCTCGTTTCTCAGTTCAGTCACAGCACAGCAGCCAAAAGAGCCTCCTGGTCTTTGACAATTGTTGTAAAGACTAGCTTTGTGTTCACCTGCATTGAGAAAACCTGGCTGCAACGGCGG	GGACCTGGGGAGTCAATATATGTGTACTGGTTATTATACAACAAAGGATTGCCACGTCTGTTGAATTGATCAAACCTGTAAGTATTATCGTGATAGAGGAAATTTTCATGATTTTCAGGACTCGGAGTATTCATATAAGTCGGATGCATATAATTGTTGGCCCTCTCGGGTTTGTTGTTTTGTCTGTACCAATGCAGCCAT	10.5
CaSNP22	*Ca-desi* -chr01	2213762	(T/C)	AACACTCCAGCAGAGACTGCGGCTCGTTTCTCAGTTCAGTCACAGCACAGCAGCCAAAAGAGCCTCCTGGTCTTTGACAATTGTTGTAAAGACTAGCTTTGTGTTCACCTGCATTGAGAAAACCTGGCTGCAACGGCGGCGGACCTGGGGAGTCAATATATGTGTACTGGTTATTATACAACAAAGGATTGCCACGTCTGT	GAATTGATCAAACCTGTAAGTATTATCGTGATAGAGGAAATTTTCATGATTTTCAGGACTCGGAGTATTCATATAAGTCGGATGCATATAATTGTTGGCCCTCTCGGGTTTGTTGTTTTGTCTGTACCAATGCAGCCATTCCGAAGAAGTCATTTGTCCGTGTGGAGGGTATCCACCGAAGAAAACCGGATATCTAGGATC	12.6

SNP IDs	Desi chromosomes	Physical positions (bp)	SNPs/SSRs	Upstream 200-bp flanking sequences/Forward primers (5'-3')	Downstream 200-bp flanking sequences/Reverse primers (5'-3')	Genetic positions (cM)
CaSNP23	*Ca-desi* -chr01	2213817	(A/G)	AAAAGAGCCTCCTGGTCTTTGACAATTGTTGTAAAGACTAGCTTTGTGTTCACCTGCATTGAGAAAACCTGGCTGCAACGGCGGCGGACCTGGGGAGTCAATATATGTGTACTGGTTATTATACAACAAAGGATTGCCACGTCTGTTGAATTGATCAAACCTGTAAGTATTATCGTGATAGAGGAAATTTTCATGATTTTC	GGACTCGGAGTATTCATATAAGTCGGATGCATATAATTGTTGGCCCTCTCGGGTTTGTTGTTTTGTCTGTACCAATGCAGCCATTCCGAAGAAGTCATTTGTCCGTGTGGAGGGTATCCACCGAAGAAAACCGGATATCTAGGATCATATCCAGGTGGTCTATATGTAGAACTCCAATCTGAGTAACCGCTTATTGGTGGC	13.6
CaSNP24	*Ca-desi* -chr01	2214019	(A/G)	GGACTCGGAGTATTCATATAAGTCGGATGCATATAATTGTTGGCCCTCTCGGGTTTGTTGTTTTGTCTGTACCAATGCAGCCATTCCGAAGAAGTCATTTGTCCGTGTGGAGGGTATCCACCGAAGAAAACCGGATATCTAGGATCATATCCAGGTGGTCTATATGTAGAACTCCAATCTGAGTAACCGCTTATTGGTGGC	AGTTTTCTTGAAATTTAGAAGCAGATAAACTAGGTTGTGCTTGTGCATTAGTAAACCAAGCAGCATTATCAGGTAATAATGGAGCAGAGGGTACTGGAAGAGAATATGTGGTAGCAGAAGAGGCATTGTAGTTTGAAGATTCATTCTTTGAGCTAATGACTGAATCTTCATTTTTATTGATGGATAGACTTGCCAAAGATG	15.6
CaSNP25	*Ca-desi* -chr01	2214038	(A/T)	AAGTCGGATGCATATAATTGTTGGCCCTCTCGGGTTTGTTGTTTTGTCTGTACCAATGCAGCCATTCCGAAGAAGTCATTTGTCCGTGTGGAGGGTATCCACCGAAGAAAACCGGATATCTAGGATCATATCCAGGTGGTCTATATGTAGAACTCCAATCTGAGTAACCGCTTATTGGTGGCAAGTTTTCTTGAAATTTAG	AGCAGATAAACTAGGTTGTGCTTGTGCATTAGTAAACCAAGCAGCATTATCAGGTAATAATGGAGCAGAGGGTACTGGAAGAGAATATGTGGTAGCAGAAGAGGCATTGTAGTTTGAAGATTCATTCTTTGAGCTAATGACTGAATCTTCATTTTTATTGATGGATAGACTTGCCAAAGATGAAGAAGCTATTTCTTCAAT	18.9
CaSNP26	*Ca-desi* -chr01	2292933	(T/C)	TGATTAACACTGTCCGAAAGGTAGTAGTAATTTTTTTTtCTTTTTTTTtCCACATGATTTTTTGTAAATCTCCTTATATAAATATTCCATGAATATTATTATTTTCTTCTCCAAGATCAACACTGCTTAGTTTTCAGACTCAAGCAGCATTATTAaaaAAAAAaaGAAATATATTGAATTTTCTGATCCCTGATTTCTTCT	CTAAAAACCCTAAGGTTCAATTAGAGAACCCTAGATTCATTCATCCCCTGTTTGCAGCAAAAGGGGAAATTTATCTCGTGAACAATTTTTTGATTCAGTGTTATATAATCGAATTTCAGTTTGGTTTTGTTTTCTCTCTGCTTCTGTAACTGTGATTTGATTTTGTTATTATTTCGTTTCTGTTCAAACACTCATGCATAG	20.6
CaSNP27	*Ca-desi* -chr01	2349214	(C/A)	CTTTTAGTAGAGACAGTATTGCACTCCATCATGAATGTTAGCTAAATTGACTTAGTATTCAATGTCAATAAAATAATGGAAGGTTCTATCAAGCATTTTGTTCTATAGTCTTGTTTACATCTCTAATTTTGTTCTTTCATTTTTGCAGGTGTAAAACGTGTTGTAGATTTATGTGCTGCCCCAGGAAGCTGGAGTCAGGTA	TGGTGATGAAATTATCACTGATCTCGCATATGAGTTGTTCAGAGTCATACAGCCATTTATGATTCAACAACATATTTCATATTTAACACAACCATTCTCTAGCACTTTAGCTTCTCAGAAATCAAAAGCAGCTTCAACTTTTTTTACCAAACACAATTTATGGCTGTGTCGAAAAGCGCTAACATAAGAAAACATAGTGTC	22.5

SNP IDs	Desi chromosomes	Physical positions (bp)	SNPs/SSRs	Upstream 200-bp flanking sequences/Forward primers (5'-3')	Downstream 200-bp flanking sequences/Reverse primers (5'-3')	Genetic positions (cM)
CaSNP28	*Ca-desi* -chr01	2349258	(G/A)	AATTGACTTAGTATTCAATGTCAATAAAATAATGGAAGGTTCTATCAAGCATTTTGTTCTATAGTCTTGTTTACATCTCTAATTTTGTTCTTTCATTTTTGCAGGTGTAAAACGTGTTGTAGATTTATGTGCTGCCCCAGGAAGCTGGAGTCAGGTACTGGTGATGAAATTATCACTGATCTCGCATATGAGTTGTTCAGA	TCATACAGCCATTTATGATTCAACAACATATTTCATATTTAACACAACCATTCTCTAGCACTTTAGCTTCTCAGAAATCAAAAGCAGCTTCAACTTTTTTTACCAAACACAATTTATGGCTGTGTCGAAAAGCGCTAACATAAGAAAACATAGTGTCAATGAGGTTGGCTCTCCAAGTAGCATTAAATGTAATTGGCACAA	24.8
CaSNP29	*Ca-desi* -chr01	2349306	(C/T)	GCATTTTGTTCTATAGTCTTGTTTACATCTCTAATTTTGTTCTTTCATTTTTGCAGGTGTAAAACGTGTTGTAGATTTATGTGCTGCCCCAGGAAGCTGGAGTCAGGTACTGGTGATGAAATTATCACTGATCTCGCATATGAGTTGTTCAGAGTCATACAGCCATTTATGATTCAACAACATATTTCATATTTAACACAA	CATTCTCTAGCACTTTAGCTTCTCAGAAATCAAAAGCAGCTTCAACTTTTTTTACCAAACACAATTTATGGCTGTGTCGAAAAGCGCTAACATAAGAAAACATAGTGTCAATGAGGTTGGCTCTCCAAGTAGCATTAAATGTAATTGGCACAAAGGCCTTGGTTGAAAACATATTTCCATGATTTCTCGTTTTGCGGCAAA	24.9
CaSNP30	*Ca-desi* -chr01	2541722	(T/C)	ATTATGAAAAACAAaCCCAAATATCAACTCCCACACTCACCTAAGTCTCCCCCAACCCACCTTCATGGCTGCTAACCCACCCTAAACCATCTTTCTTTAAGATTAGTTCTTCCACCATTAAAGATCCATTGAACCCTAACCTCCAAAAGCATGACTAAGCTCCACCATCAAAGATCCACTGGATCTAACACCCAAAAGAGT	GAACCAACCTCATAGAATGCAGCTGCGAGAACCATAGAAGTTCAAGCAGTGGGATAACAACTATCATCGCTCATTTGCAAGAACAACTTGAGAGCATAAATGCGAGTGGTTCATCATGAATGAAGTTCACTAGATGCAAGAGATTCAAAACAAACCTTGCTTCTTGTGGGGAAGCACACGACTTGATCATGGAGTTTCCAA	25.4
CaSNP31	*Ca-desi* -chr01	2553120	(A/C)	GTGCCAGCAAAGCTTTTTGGAAGAATTGGAGAGGACTGTTGCACTGCTTGCCTTTGAAGATGTTTCTAATTGTCCTGTTGGAGAGCTTCTCGACATATCACAGCGTCTAAAAACAGCAAGCGAGGTGAATGCAGCTATACTTACAAGCCAGAGTCATGAAAAAGGTTTGTGTTTTCTTCATAAATCTGTTTGTTTCTATAT	TAACTTTGGTATGTGAATGGTTTTAAATACTGTTTTGCTACTTAGTTTATCTAAGAAATGTGTGTAATTTTtATGCATTGCTGCATGTTGTGTGGTGATTAAGATATATTATTTATATATATATGTTTATTGAATAGCTTGTTGTGTTGTAAAATTTGGGAACAAAATAGAAGCTTTGCCAGATTCACCCGATTAAACAAC	26.8
CaSSR4	*Ca-desi* -chr01		(GAA40)	TTGACGCAGAGAACTCTCAA	ATTGGTGTGATGGGTGGATT	28.9
CaSNP32	*Ca-desi* -chr01	2553150	(C/T)	GAGGACTGTTGCACTGCTTGCCTTTGAAGATGTTTCTAATTGTCCTGTTGGAGAGCTTCTCGACATATCACAGCGTCTAAAAACAGCAAGCGAGGTGAATGCAGCTATACTTACAAGCCAGAGTCATGAAAAAGGTTTGTGTTTTCTTCATAAATCTGTTTGTTTCTATATATAACTTTGGTATGTGAATGGTTTTAAATA	TGTTTTGCTACTTAGTTTATCTAAGAAATGTGTGTAATTTTtATGCATTGCTGCATGTTGTGTGGTGATTAAGATATATTATTTATATATATATGTTTATTGAATAGCTTGTTGTGTTGTAAAATTTGGGAACAAAATAGAAGCTTTGCCAGATTCACCCGATTAAACAACCACTATCCCcTGTGTGCCTTGTGATGGTTT	30.5

SNP IDs	Desi chromosomes	Physical positions (bp)	SNPs/SSRs	Upstream 200-bp flanking sequences/Forward primers (5'-3')	Downstream 200-bp flanking sequences/Reverse primers (5'-3')	Genetic positions (cM)
CaSNP33	*Ca-desi* -chr01	2553187	(A/G)	AATTGTCCTGTTGGAGAGCTTCTCGACATATCACAGCGTCTAAAAACAGCAAGCGAGGTGAATGCAGCTATACTTACAAGCCAGAGTCATGAAAAAGGTTTGTGTTTTCTTCATAAATCTGTTTGTTTCTATATATAACTTTGGTATGTGAATGGTTTTAAATACTGTTTTGCTACTTAGTTTATCTAAGAAATGTGTGTA	TTTTtATGCATTGCTGCATGTTGTGTGGTGATTAAGATATATTATTTATATATATATGTTTATTGAATAGCTTGTTGTGTTGTAAAATTTGGGAACAAAATAGAAGCTTTGCCAGATTCACCCGATTAAACAACCACTATCCCcTGTGTGCCTTGTGATGGTTTATACCCGAAAAATCGATTAAAACTTATAACGCTTGTT	32.5
CaSNP34	*Ca-desi* -chr01	2554326	(T/G)	AGCTCTAGAGTTTGCTAATAGTATTTTTGTTCTTCCACCAGACCCAAAACTTCCAAGCTTGTTGAAGATGCTGTTATGGGCCCAAAACCAGCTGGATGAGAAAGCTACTTATCCTCGCATAAAAGATCTATCCACGGCTGCATTAGAAGATCCTGTTTAAATTCTTGTAACATTTTAAATTCAGTTTGGAGAATTTTAGGT	CTAGATGAAGTGATTGTGGTCATTGGTGTTAAAAATTGTGTATTACTTTGATCGAAAGATGTTTGATAACATTGATGTTCGTTTTATCCTAGATAGCTGCTCCAGTGGTTTATACCGTTGTACAAAAAGATTGTCAATACAGTTATTATCCATTCATTTTATCTTCAAGTTACCGATTATTAACATATTCTGTTGCAACTT	33.6
CaSNP35	*Ca-desi* -chr01	2555171	(C/G)	AGAACATAAAAAaGGCTGCCTCAAGTCTTGATCCAAATGACATAAACCCAGGTTGTGCTGTCAATGTCTTTGAACTCTTGAACAGGTGCATCTGCAATGAATTTCTTTGGCATAGTATCTCTAAGATAGGAAGAGGAACATCCATCATCAAAGTTAAGTGCATAGCTTGAAGGATCATATTGAAATTTTCGCCTCTGTTTG	CTCCATTGTTCAAAGCCCTCTTCACTGCAGCTCTCATCCTCCAAAACAAGCTCCTACAACGTTTCTTTCCATAACCATTCACCCATTCGAAGTTTTCCAACAAACCCATTTTGTCAAAGAAAGAAAGTTACAAACTTTGAGCAATTTCCTCAATGGGTATTTATAATGAGGTTGAAAACTTTCTGATAGTAAACAAGTGAT	34.2
CaSNP36	*Ca-desi* -chr01	2573180	(A/C)	GATATGACAATTAATTTTTTCCACCTTAGTTTGGTTTAATATTAGTCTAAGTGTGTTTGAATTTGTTCCAAGTCACAATGTGGTTTAATATTTTCCATTGTTGGGCTTTTGTATCATTTTACTATGTGATTAAGTTGGCTTTGGGCTGCTAAGCCCAGAACCAGAAGACAACTGCTCTTCACAATTTAATTGAATGAGATT	TTTTCTCTAGCATAGCATGTTGTCTCAAAAGCTTATAATTTTTGTCATGTTCAAATTGATCAATTTAAATGTATATTTGAAAACACAACGAGAATAATGAGTTGAACTTTTTCTTATTATTTGAGTATTTGTGAGAATGACTGAATGACATACAAGACTGTGGGTAGCTGCTAATGCCTTACCACAAGCTAAAGGCAATGC	35.8
CaSNP37	*Ca-desi* -chr01	3953451	(C/T)	CCATCTAGGAGATTCAGGAATGAAGAACAGACCAAGCAGCAAGCAAATGCAAGGCACTAATCCTTCAAAAGTTCAAAAAGCCAAACCAAATCAACAGTTAGATATCTTTCTCCATAATCTGTTACCCTTCAATTCAAAATTAATTGTTATATACTAACTTACCAGCTAGTGCTAGTTCTCTCCAATTTATGACACTTCCTA	TAAAAATGACATTGATGATCCAATGACAATCATAAGCTGCAATACAATTATAATCAATACTAATCCAATTAAATGATACTTATTAGTCAAAAAaTAGAAAATTGTAAAAAAAAaTCTCACCTGATTTGTTGTTGCAAGTCCTCCTCTAaGATTTTTTGGTGCTATTTCTGCTATATATACTGGAACCTGATCCATTTTGCT	36.7

SNP IDs	Desi chromosomes	Physical positions (bp)	SNPs/SSRs	Upstream 200-bp flanking sequences/Forward primers (5'-3')	Downstream 200-bp flanking sequences/Reverse primers (5'-3')	Genetic positions (cM)
CaSNP38	*Ca-desi* -chr01	3963327	(T/C)	TAAAGAAGTTTGATTCCTAAAAAACTGTATAGATTTACTATGCAATTTTCTGTGATGCTATTAATTGTTTTTCACGTCTTGTTTTATGTGCAACACTTGATGCCAGTGATGATCAAAATCATTTGATAACTTCATGCAGCATAAAACGTTAGCTACTTAACCCAAGATGGGATCTATGTTGTTAATTTTGGATAGTAGCGC	GAACAGCAGACTCTAAATCTGGGATAGCAGTTTAGCGGAAAGCGGGAAAGCAGCTATTCTAATATAATTTTATTTGAAAATCCATTCTCTAAACCCAAAAAaTTGAAACTTCATCTTCTCCAAAAGGCATGCAGTCTCTTCTCAACATATGAAGGTGGCGGCAGAAAACAGCAGACTGTGGCCGAAAAGTTTTCTTCCTAA	37.8
CaSNP39	*Ca-desi* -chr01	3995579	(C/T)	GATCTTTGACTTTCTATATTCATGACAGGTATATGTAACATGGTTTAATTTTGCAGGAGTTACCAAGTTTGATCCTTCTACCGGAAAACAGTTCAAGGCCAAGGCTCCCACCCAGTCATACACAACATACTTTGCTGAGGCTTTGATTGCAGAAGCAGAAGCTGACAAAAACATTGTTGGAATCCATGCTGCAATGGGAGG	GGAACTGGCATGAATCTCTTCCTCCGCCGTTTCCCTGCAAGATGCTTTGACgTGGGGATAGCAGAACAGCATGCTGTTACATTTGCTGCAGGTCTGGCTTGTGAAGGGCTTAAGCCTTTTTGCGCAATTTACTCATCCTTCATGCAAAGAGCTTATGACCAGGTAAGATCTGATAACAACTCTCCCAAGTACATATATCCT	39.4
CaSNP40	*Ca-desi* -chr01	3996356	(C/T)	TAACTTTAGTAAGTAATGCTTTAAGTATATATTTCATGTCTATTCCAAGGTGGTGCATGATGTGGACTTGCAAAAGCTGCCTGTTAGATTTGCAATGGACAGAGCTGGATTAGTTGGAGCAGATGGTCCAACACACTCGGGTTCTTTCGATGTCACATTTATGGCATGTCTCCCTAACATGGTGGTGATGGCTCCTTCTGA	GAAGTGGAGCTTTTCCACATGGTTGCTACTGCAGCTGCCATTAATGATCGACCTAGTTGTTTTCGATATCCAAGGGGAAATGGAATTGGTGTCGAaCTACCAAAAGGGAATAAAGGCACTCCTCTTGAGGTGAGTTAGATTTATTATGGCCATCTTTATACTTCACAATTATATTCTTATGAGGATGATTAGGAAGAAGTG	40.7
CaSNP41	*Ca-desi* -chr01	3996916	(C/T)	TGTATTGGTAATATTTCTCTTGAAAACAGTTTCGGCAAACAAGATTAACATTTGTATTTCGACATCTTAACAGATTGGAAAAGGTAGGATTTTGATTGAAGGGGAAAGAGTGGCCCTTTTGGGCTATGGATCAGCTGTTCAGAACTGTCTAGCTGCAGCTTCCTTGTTGGAGCATCACGGCTTACATGTAACAGTGGCCGA	GCACGTTTCTGCAaGCCATTGGATCGTTCCCTCATCCGCAGCCTAGCCAAATCACATGAGGTTTTAATCACTGTGGAAGAAGGATCAATAGGAGGATTTGGGTCTCATGTGGTTCAGTTCATGGCCCTTGATGGTCTTCTTGATGGAAACTTAAAGGTATATATGACCTTAAATATTTGCATGTCTTATAATACTCCACCC	41.8
CaSNP42	*Ca-desi* -chr01	5004855	(A/C)	AAACACATTTACAGAATAGATGTAGTCACTACCTTACCTCATTCTCTAGTTCTAACCTTCCTCATCTCTCAACTAGTATTATACATTATATGATAATGCAAATAATGTTAATAAGATTTGTGTAATATTGTGATTAATTCACACCACGGCGACAGTAACATAACACCTGTAATAGCTGAAGCTGCAAAGTTGTTCTATGTA	AGTATCTAAATTTAGTAAATTAAATAATGTGGAGAGAATATAATGTATTCTACACTATAATCTCACAGTGCACTATAGAATTAACCAGTGTGTGATGGTGGAAAAATTACCTGCAGCTCCAGATTCTAAATTATCACCACTGGCAGCATTCTTTTTtCTCCTTGACCTACACACCCAATAAAATTAGCCACACAAAACAAT	42.5

SNP IDs	Desi chromosomes	Physical positions (bp)	SNPs/SSRs	Upstream 200-bp flanking sequences/Forward primers (5'-3')	Downstream 200-bp flanking sequences/Reverse primers (5'-3')	Genetic positions (cM)
CaSNP43	*Ca-desi* -chr01	5004917	(C/A)	CATCTCTCAACTAGTATTATACATTATATGATAATGCAAATAATGTTAATAAGATTTGTGTAATATTGTGATTAATTCACACCACGGCGACAGTAACATAACACCTGTAATAGCTGAAGCTGCAAAGTTGTTCTATGTAAAGTATCTAAATTTAGTAAATTAAATAATGTGGAGAGAATATAATGTATTCTACACTATAAT	TCACAGTGCACTATAGAATTAACCAGTGTGTGATGGTGGAAAAATTACCTGCAGCTCCAGATTCTAAATTATCACCACTGGCAGCATTCTTTTTtCTCCTTGACCTACACACCCAATAAAATTAGCCACACAAAACAATTAAATACATCATAGAGCAAAATCACCACAGCATTTAACTGCATCTATGATCAAAGTCATACA	43.6
CaSNP44	*Ca-desi* -chr01	5022262	(G/T)	GCATCTGATAAAATTGGAACGATATAGGATATAAAGTATGTTATGTTAGTAGCTAAAATTTATCTATTAAAGAAGTGTTTCTTTAATAAATGTGTTTTTAAGTTGTTGGGTAAGATCATAACTACCATACCCATTTTCTAATAATTGCTGTGGTCACTCAGTGCCAGCAAGCAACAACAAACTTAATAAGCAGCTGCAAAT	GTGACACCCCTCAATCATGATACTAGTATAAATACTATGCCTTATATATGGTGAATGATTATGTTCCAATATCAAAGTATCAATTAAGTAGGGTTATAGAGATTAAATGGTCCCACGGTACCAGTTATATTTCTTAACAACACAAACTTGAGGCTAGTATCCAAACAGACCCTACTCTACACAGAGTATACATGCTAGAAA	44.8
CaSNP45	*Ca-desi* -chr01	5039614	(C/A)	AATTATATTTTTAGTGAGGTTGAATTTGTAGAATGAATATCATTTTTCTTTTGTAGAAAAAGTATTAGCAAAAAATAGGGGTACGAATAGCAGCGTGAAAATATATTCGATGGTGTGGGGTATTAAAGTTTGAAGCCCAAGCGAGAAGTGAAAAAAGGAGGCCCAAAGGAGAAATGAAACAGAGAGAGAAAGTGACAGACA	CAGCAGAAACAAAGAGTTAGTGTTGTTGTTGAGGTTGTAGTGAGATTAGTGTTACAGCAGCAACGATCCATAGAGAAGATTGAAAAGATGAACACAGATAtCACTGCTTCCACTAAACCAGAGTACCCCGTCGTTGATCGCAACCCTCCTTTCTCTACTGTCGTCGGCAACTTCAATACCCTCGATAACCTCCGTTTCGTC	46.9
CaSNP46	*Ca-desi* -chr01	5061066	(T/A)	ATTGATTAAACTAACTGTCTTACTTACAAACTCTAGATCCTTTTGTAAACTCTCTTCACAGGTTCCGTGGAAGAAaGAAAAAaGATACTAGTTGGCTACCACCCAATAGCTTGCCAAATGCAGAAACTTGGAAGTGTATGGCTAACCTAATAATGAAAATTGTGCAGCATAGAAGGAACATTATTCCCAAGGAGCTGAATA	TGTACATACTAGGACAGCACAACGTCTTGTTCCATGTGCTTCAGTGTCCTGTTGCAAACAACTCAGCAGCTTTCCTTGCAAACGGGAACATGTTTGAATCCCTAAAGAAATTGTGTATAACTCATCAAGAACATCGCAAATTGTCTCAACCAAAACTTTAATCAGCAGAAATTTATATCCCTTGTTTTCTGCGGCGTAAGT	47.8
CaSNP47	*Ca-desi* -chr01	5061125	(G/C)	AGGTTCCGTGGAAGAAaGAAAAAaGATACTAGTTGGCTACCACCCAATAGCTTGCCAAATGCAGAAACTTGGAAGTGTATGGCTAACCTAATAATGAAAATTGTGCAGCATAGAAGGAACATTATTCCCAAGGAGCTGAATAATGTACATACTAGGACAGCACAACGTCTTGTTCCATGTGCTTCAGTGTCCTGTTGCAAA	AACTCAGCAGCTTTCCTTGCAAACGGGAACATGTTTGAATCCCTAAAGAAATTGTGTATAACTCATCAAGAACATCGCAAATTGTCTCAACCAAAACTTTAATCAGCAGAAATTTATATCCCTTGTTTTCTGCGGCGTAAGTTACGCATTTATCAGTTTCTACTTAAGTCAGGAATATAGCCTGCATCCTTTAATATAGAG	48.9

SNP IDs	Desi chromosomes	Physical positions (bp)	SNPs/SSRs	Upstream 200-bp flanking sequences/Forward primers (5'-3')	Downstream 200-bp flanking sequences/Reverse primers (5'-3')	Genetic positions (cM)
CaSNP48	*Ca-desi* -chr01	5498890	(A/G)	ACAACTGATGGAAATTAGCTGAAAACAACTTATGAACAAGTCGTAAGAAGTTTCCATAAAATCTTCCAAACAGGATAACAAGTGCTTAGGCCAATAGATAATCATAAGCCAATCCAAACAGACCCATATTTTGGACGAATGAATGCTGCGAAAACTGTGTAAAGCTATTTGAGTATTTCAACTTTGAATGAGAAATAATGG	GGGAAAGAACAAAGTGAAACTAGAAGGAAAAGGAAGAAATTAGTTGCTGCGAGTTTCAACCATTTGTTTGTGTCTTATTGTCTACTTTTTCAATTAGTAAAGGAATCACTTACTGTAATGAATCCTGGAATTTGTTGAATCGTATAATATTGTGCTAGTGAGAGTGTGCATTTCTGGATGAATGTACCATTTTCAATTTTG	50.5
CaSNP49	*Ca-desi* -chr01	5511162	(T/G)	TATAAAGGATAAACAAGAAAAaTTAACAGAAATTACTGTGTTTTTTCAATTGATTTTGGAAGATTTTTTACTACATTTTCTGAAGCCAaaCTAGCTGCTTATTTTGACTTCACCACCTGCTTGTTTAGCTGGTAGAAGTGCTGTATTTTATAACCGCTTGTTTTCAACTGTGCATTTCTAGTTGCTTGTTTTGACTTCACA	TTTTTAGTTTGCATATATCTTAAGTTCTCCGTGCTGCATCTGTTCTTATATATGTGATTTTGCTTTCTGACCGCTATTGGCTTAATTATTAGTCACTTTCTACCCCTATTTAAACCCTAGAGGCAAATTTCTGAAGCTCTCAATTCTCTTGTTTTCCTTAATCTCTAATTAAAGTTCTCATATTGGTGTATTGATTTTTGT	51.8
CaSNP50	*Ca-desi* -chr01	5537208	(C/T)	AGCAGAGTTAGAACAAGTGAAGAAAGAACAAGAGGAACTAAAGGAGAAAGAACAAGCAGCAGAAGCTCTAGCTGTTAACCTCACGGATGAACTACAAAGTAGCAAAAAAAaTGAAGATTCTCTTGATAACAAAGAATCAAATGAGAGTTATTGTCAAGACATAGACTTGAAAATCAAGCAGCTATCGTTCGAGAAAGAAAA	GCGAAAAAaGAAGAAGAAGAGATGAGAATAAAAGCTCAGGAACTGAAACAAGAAGCTGACAAATCAAGAGTTTTGTCTGAAGAAATAGAAGGGAAACTAGAGCTTTTACTGAAACAAGCTGAAGAAGCAAAAGCAGCTGAAAAAaGGGCTGTTGAGGAGATGAAGTTGTTGTCTGATAACACACAAGGTAGAGTCTCAGTT	52.6
CaSSR5	*Ca-desi* -chr01		(GA11)	GTTGAGCAACAAAGCCACAA	TTCTTGTCTGGTTGTGTGAGC	53.8
CaSNP51	*Ca-desi* -chr01	5537508	(C/T)	AGAGCTTTTACTGAAACAAGCTGAAGAAGCAAAAGCAGCTGAAAAAaGGGCTGTTGAGGAGATGAAGTTGTTGTCTGATAACACACAAGGTAGAGTCTCAGTTTCAGATCCAAGTGGTAAGATCATATTGACAGTTGATGAATTTGCTGCACTAAGTGGTAAAATCAAGGAATCTGAGGATTTGATTGAGAGGACGGAAAC	GCTGCAATGGCTCAAGTGGAAGCAATCAACACAAGAAGAATTGAAGTGGAGAAAAAAGTGGAAGCTAATCTTAAAGCAATCGAAGAAaTAAAGGCGGCAACAGATATGGCTTTGAGGAATGCAGAGATGGCAGATTCGGCGAAAGTAGCAGTTGAAGGCGAATTAAAGAGGCGGCGCCAAGAAGAACAAAAAGGTGTATCT	55.4
CaSNP52	*Ca-desi* -chr01	5568121	(A/C)	GGCAATAAAAAGACATCCTGGATTTTCACGTATGCAAAGGGTTCCATACCTGTTAGATGAAATTTGAGATATCAACATATGAATCAACTAAAAGAAAGTAATTACATAATTGTGGACTGAATTCTTAAAACAATGTTTTtGTTTAATCAGAATTTAAGAGGATGCTGCCATGAACAAATATATGATTTTGGTGATTCGTAT	AACCTATTTTGTAGTGAAAAATTGAAAAAaCTATCTATTCACCTACAGCATGCTGCTTACTAAGTTAATCACTTTAAGAAAACTTATTTTGCTTCGGGTTCATTTAACTGTCGAAAATCTGCTTTAATGCATTATTAATATTAATATTCTGAAAACAGGTGATTTGTTCACTATAACATTTTAGAGAAGCTTTAAAACTTC	56.7

SNP IDs	Desi chromosomes	Physical positions (bp)	SNPs/SSRs	Upstream 200-bp flanking sequences/Forward primers (5'-3')	Downstream 200-bp flanking sequences/Reverse primers (5'-3')	Genetic positions (cM)
CaSNP53	*Ca-desi* -chr01	5613829	(C/A)	TGCACAAAAATCTCAGTATATTAAAAAGTGAGCAATT<br>TTTTAAAGAGTAAAATAGTACTAAATTTTAATTGTTG<br>ATGAAAAAaTTAATGGTTCATACATACTGTAACACAT<br>TTATATCTTGTTATGTATCTGCATATAATATCAAAGTT<br>GATTTATGTAGCAGCAATAATTGAGATTACTTATTTT<br>ACCTTTAAAGTGAAT	AGTGAATTCTATTAACTGTAAAAGGTTCACTTTTAATCA<br>AACATCACAAAGAAGTTACACTGTCGGCACAGCTGCAA<br>GTGCAGGGTTTATTGTAGCATATGTATCATAAAGACAT<br>TGATATAAGCATTGCATTTTCCCTTTATCAGTTTCAGGG<br>TTTACCAAACAATATTTATAACGCGACCTTATATTTCAC<br>CACACATG	58.4
CaSNP54	*Ca-desi* -chr01	5644131	(G/C)	GTGTTTTTGCACTCATTGTTATCATTAATTTATAATAC<br>AACTCAAGATTTGATTAAGCTCAAAGTAACTGTTATA<br>TCATTTTTCTGTGAAGGCTGTCGCTGCTGCACGTTCAT<br>ATGGAGGCCGATGATAAGATGTGGCAAAGTATGTTG<br>TGAGGAAGGATGTTGCTAACTTAAGCATTACAAGGT<br>TGAAGAAGACCCCTTA	AAGTTCTATATATGTTTCTCTTCACAAAGAAACATAAAA<br>TACTATGCTGCTAAATAATAATTATGCAGGAAGCCTGA<br>TAAATAGGTTCTTTTGCATAAAAGTTTGTAGTGTTATTG<br>ATAACTTGGTATAATTGTACTTTTAATATGATATGTCCA<br>TTCCCAAGAGAGATTTCAACACAAAAACCACTTGAAGA<br>CTATGAAG	59.5
CaSNP55	*Ca-desi* -chr01	5676601	(T/C)	AAAATGACCTAATTGTTCCTGGTCCAATGTATATTATT<br>TATTAAAATAATCATAGAAAGTCAAATTTTAAGAAAC<br>CTGGGAaTAGTTTTGGATAGCTCTGTTGAAACCACTG<br>TGATAAGATTGTACAACTTCATCCACAACTTCCTCGAT<br>AAGATCACTTTGCTCCTTCAAAAATTGGGCAGCCCCA<br>TCACGGTCTTTTGA	GTCAAAATATGAACAACATGAGGTAACGAATCAAAAC<br>GAGCAGCAATCCAAATCTCATCTATCCTAGAGATTTCTT<br>CTCTCAGATACTGCATTCAAATGTAGAACAAAGACTCA<br>AATTATCACGAATAACTAAACGAGTATTCAGTGCAACG<br>CATGAAAAAGTACAATATAAATGCAATTCCGTCCATCCT<br>ATAAGTTTGC	60.2
CaSNP56	*Ca-desi* -chr01	5753424	(G/A)	GGAACCTGACATACATAATCACAAAACGTTCATTAAA<br>ATATTTGAGAAATAGTAATGAATTTTTTTtCtAGAACA<br>TGTGTTTTTTtAGTACTTACAATTCCCTAACTTTTGTAA<br>GATTGTTGATGTTTTTAGGCAAAGGTCCACCTAAAAA<br>aTTGTGATCCAAGCGCCTGCAGCCGCAGAATTATACT<br>TCAAGACCAAAGC	AAACAAGAAATTTGTATTATCAATTTATATGAGTTTTGG<br>ACACTCACACTACTTCAAGGTGCTGGACAAGTCCAAGT<br>GTTGATGGAATGCTGCCAGTAAATTGGTTACTTTCAAA<br>AAGGCTGCAAAAAaGTAAaTGTAAATTCATGATGAAAA<br>AAaTTCTACTCAAATTTGTTTAATAATGTAAACAAATTCT<br>TGATGAAA	61.3
CaSNP57	*Ca-desi* -chr01	5753484	(T/G)	TTTTTTtCtAGAACATGTGTTTTTTtAGTACTTACAATTC<br>CCTAACTTTTGTAAGATTGTTGATGTTTTTAGGCAAA<br>GGTCCACCTAAAAAaTTGTGATCCAAGCGCCTGCAGC<br>CGCAGAATTATACTTCAAGACCAAAGCGAAACAAGA<br>AATTTGTATTATCAATTTATATGAGTTTTGGACACTCA<br>CACTACTTCAAGG	GCTGGACAAGTCCAAGTGTTGATGGAATGCTGCCAGTA<br>AATTGGTTACTTTCAAAAAGGCTGCAAAAAaGTAAaTG<br>TAAATTCATGATGAAAAAAaTTCTACTCAAATTTGTTTA<br>ATAATGTAAACAAATTCTTGATGAAAAAAaTTGGAATG<br>AATCTTACACATGTATCAAAGACATTTGAGAGCTAAAA<br>AGTTGAGGTG	62.5
CaSNP58	*Ca-desi* -chr01	5776369	(T/C)	ACTAATTGTGTAAAGTTTGATTTTTGATGAAAATGGG<br>TCTTTTTATTTGTTATCTTAATTTGATTTATGTTTGTGG<br>GTTTATGCAGCTGGGAAACTTCCAAAGCATAACAAT<br>GTTTCTTGGAGGGGGACCTCAGGTATGCAAGATGGA<br>AAGGGTGATGGTGTTTCTGCTGCAATCAAGGATCTG<br>GTTGGTGGTTATTATGA	GCTGGTGATGCcATCAAGTTTAATTTtCCTGCTGCTTTTt<br>CCATCACCATGTTGAGTTGGAGTGTCATTGAGTATAGT<br>GGTAAGTATGAAGCTACAGGGGAGCTTGATCATGTCA<br>AGGATATTATTAAATGGGGTACTGATTATTTTCTCAAGA<br>CATTCAATAGTACAGCTGATACCATTACCACCATTGCTG<br>CACAGGTA	63.8

SNP IDs	Desi chromosomes	Physical positions (bp)	SNPs/SSRs	Upstream 200-bp flanking sequences/Forward primers (5'-3')	Downstream 200-bp flanking sequences/Reverse primers (5'-3')	Genetic positions (cM)
CaSNP59	*Ca-desi* -chr01	5807642	(G/A)	TGAGCACAGCTGAAGCAGCATGAACCTCTACACGAAGGTTCTGAAAATCATCCATCGCAGCAGCTAGGGCTGGCAACAAACACCTCCTGATGATATTGAACTTGCAAATCTGGACCCAAATCACTAGACAGTTGCCCAATTGCATTAATAGCTGCCCACTTTACACGAGGATGTTGATCAGGAAATGAATTCAATACCATA	CCACCACTTGCTCCAAATTTTATATCATGACCTTTGAGCATCCTTCAGCTATCTGAGCACGTGCAATCAGTGCAGCATGACGTTTTTGCCACTCAGGTGCAGCCAAATATGCAGGTAATTGTTCAGAAGCAACAGGCACAATCGTATTTCCTCCAAAGATATAGATAACCTGTCTAAACATTCCTGTCCAACACTATAATT	64.7
CaSNP60	*Ca-desi* -chr01	5823260	(C/T)	CTTCAACATTCAAAACAGTAACTAGTAAGAAGTAACTGGTGATCCAAAAGAATGAGCATGTTCATTGAAGCCCTTAACACCAATCCAGCTATTCCCAGCTTCCTTGACCAGCCCTTTTGTCCTCATGATGCTTCTCAGTTCATCAACACAATCCCACCTACCAATGGCTGCATAAAAGTTTGATACAAGCACAATATTGCT	GCTTTGTCGGGTTCCAACTGCAAGAGATGCTTTGCAGCAAATTCACCTCTTTCAAAATCTTGATTAAGACTGCAAGCATTCAACAATGCTATCCATACTCCTGCAGTAGGTCTACTACCCATTTCAATCATATTGTGATAAGTGGACCAAACATCTTCTATATTACCGGCTCGACCTAAGATATCTATGAAGCACGCGTAG	65.9
CaSNP61	*Ca-desi* -chr01	6080870	(T/C)	GAGGTGCAGATCTGACCATTGCAAAATTACGCAATCTATGCAAGAAGAAACAACCAGAGGATTGAATGGTAATTTTCTTAAGCTGCAATTCCAATTGTTTAACATGCTCGTTTGTATGAATCAAACTTTGTGATGTTGCTGAATAGTATCCCTtACTTGTGCTAGAGTTTTGTATCAATAGCCTTGCCCAAAAAATTTGCAA	AAGTGCAAAATATCCACTTTGCTCTGGATTTGAAGCTTTATGCTGCCAAACAAAAGGAATGGGCCTCTAGAGAGGGAGTTTAACTAGGTGTCTTCAGAGCAATGTTTGCTGGTACGATTTTTCTACCATTTCGTGTTGATTCTTGAGGCACAATAGTAACGCATGTGATATCTGTAGTCTTTCAACAGCATAAGAAAAGAC	66.7
CaSNP62	*Ca-desi* -chr01	6083987	(T/G)	AAAGACTTGCATTGCACTGGAGAGATAAGTATATCTCCCGGAGAAACCTGCATTTTATATAGATTCTCACTTTTCTTTTGGACAGAAACATCAGCACAAGCACAAATATCACACACAaTATTGTACCTCCTCCCACGTGCTTGAAGCCAGTGGATCCACAGAAACTTCTCTATCTGTATACTGAGAAGATGTTGCAGCACT	GTTTTGTCTATGAGAGACAATTGAAGTATTCTTTCAACACGATCTGGCTTCTCATCCAACCGTATAGCAGCCATATCCGATAGAAGCTTTAGGGACTGTTTACACGGCAAAAGATATTTTACATGGTGATTAAAAAAGTGTTGCTTCCAATATAATTTCTTCAAGTATGAGCAAGTTGAAAGATTATCATTGAACTAAGTA	67.8
CaSSR6	*Ca-desi* -chr01		(TA30)	TCATTAAAATTCTATTGTCCTGTCCTT	ATCGTTTTTCTAAACTAAATTGTGCAT	69.5
CaSNP63	*Ca-desi* -chr01	6108008	(T/G)	CTCAGGTACATGTATCCTTCTCACTCTCTCTCATTAATTGATTTATTCACACTTTCCCCTAGGTTATCAATATAGGCAATTAGCGCCGCAATCATGCCAGTGCGGCATGGAATAGAGGCTTGGGTGTTGCACCGGAGTTTGCAGCAGCGTGGTGCAGTTATTTTCATGTCTGGAGTGGCACAACAGACCCACTGCAGCGTA	GGTTTCGCAAAAAaCGTAACTTTCACTTTCCTTGTCTATTATGTGCAAACCAAAGCTTCTCTTTCCTTCAAAACTTGCATTCTTGCGAGTAGAACAAGAACAACGACCTAGCTGCCTCCGTTGCAGGCGCCATCGACCCCACCGTCGCATCTATCCTCTCCGAGCAGCTCTCTTTCTGTGCGACCTTGTATGTATTTCTGC	70.4

SNP IDs	Desi chromosomes	Physical positions (bp)	SNPs/SSRs	Upstream 200-bp flanking sequences/Forward primers (5'-3')	Downstream 200-bp flanking sequences/Reverse primers (5'-3')	Genetic positions (cM)
CaSNP64	*Ca-desi* -chr01	6108016	(C/T)	CATGTATCCTTCTCACTCTCTCTCATTAATTGATTTATTCACACTTTCCCCTAGGTTATCAATATAGGCAATTAGCGCCGCAATCATGCCAGTGCGGCATGGAATAGAGGCTTGGGTGTTGCACCGGAGTTTGCAGCAGCGTGGTGCAGTTATTTTCATGTCTGGAGTGGCACAACAGACCCACTGCAGCGTATGGTTTCG	AAAAAaCGTAACTTTCACTTTCCTTGTCTATTATGTGCAAACCAAAGCTTCTCTTTCCTTCAAAACTTGCATTCTTGCGAGTAGAACAAGAACAACGACCTAGCTGCCTCCGTTGCAGGCGCCATCGACCCCACCGTCGCATCTATCCTCTCCGAGCAGCTCTCTTTCTGTGCGACCTTGTATGTATTTCTGCCTGGTTTT	72.5
CaSNP65	*Ca-desi* -chr01	6108035	(T/A)	CTCTCATTAATTGATTTATTCACACTTTCCCCTAGGTTATCAATATAGGCAATTAGCGCCGCAATCATGCCAGTGCGGCATGGAATAGAGGCTTGGGTGTTGCACCGGAGTTTGCAGCAGCGTGGTGCAGTTATTTTCATGTCTGGAGTGGCACAACAGACCCACTGCAGCGTATGGTTTCGCAAAAAaCGTAACTTTCAC	TTCCTTGTCTATTATGTGCAAACCAAAGCTTCTCTTTCCTTCAAAACTTGCATTCTTGCGAGTAGAACAAGAACAACGACCTAGCTGCCTCCGTTGCAGGCGCCATCGACCCCACCGTCGCATCTATCCTCTCCGAGCAGCTCTCTTTCTGTGCGACCTTGTATGTATTTCTGCCTGGTTTTtAAATTTtCCTCTGTTTTG	74.3
CaSNP66	*Ca-desi* -chr01	6108094	(C/T)	CGCAATCATGCCAGTGCGGCATGGAATAGAGGCTTGGGTGTTGCACCGGAGTTTGCAGCAGCGTGGTGCAGTTATTTTCATGTCTGGAGTGGCACAACAGACCCACTGCAGCGTATGGTTTCGCAAAAAaCGTAACTTTCACTTTCCTTGTCTATTATGTGCAAACCAAAGCTTCTCTTTCCTTCAAAACTTGCATTCTTG	GAGTAGAACAAGAACAACGACCTAGCTGCCTCCGTTGCAGGCGCCATCGACCCCACCGTCGCATCTATCCTCTCCGAGCAGCTCTCTTTCTGTGCGACCTTGTATGTATTTCTGCCTGGTTTTtAAATTTtCCTCTGTTTTGTGAGAGTAAAACAGAAGAGCCACACTAAAGCCTCATTTTTTTtGtACTTTTCCCCGTCC	75.6
CaSNP67	*Ca-desi* -chr01	6108140	(A/G)	CGGAGTTTGCAGCAGCGTGGTGCAGTTATTTTCATGTCTGGAGTGGCACAACAGACCCACTGCAGCGTATGGTTTCGCAAAAAaCGTAACTTTCACTTTCCTTGTCTATTATGTGCAAACCAAAGCTTCTCTTTCCTTCAAAACTTGCATTCTTGCGAGTAGAACAAGAACAACGACCTAGCTGCCTCCGTTGCAGGCGCC	TCGACCCCACCGTCGCATCTATCCTCTCCGAGCAGCTCTCTTTCTGTGCGACCTTGTATGTATTTCTGCCTGGTTTTtAAATTTtCCTCTGTTTTGTGAGAGTAAAACAGAAGAGCCACACTAAAGCCTCATTTTTTTtGtACTTTTCCCCGTCCAGCCATGTTAGCTTCATCTCATGGTCCACAAAGTAACAGCACATCG	76.9
CaSNP68	*Ca-desi* -chr01	6111677	(A/G)	AGAAGAAGCAGAGCGACTCTAACGAAACGAAGTTATGATCGATCAACGGAGTTAGAAAATGTATGGTGACTTCCTAGATAAGGTCATTGTGCCTAATAGGGCTGCCAGATCCAACCTGAGAACTAGATAACCAATTTGCCATTGGACTTCCTGGATAAGACAAACCAGTGCCATACGCAGGATTTCCATATGACTGGAACC	CCGGATTTACCACCCATTGGAACAATGTTTGGTGACTTCTGAGGTGAAAGCAGCTATCCAAGATAAGCTTTCTAAAGCTCAAGTAAATTCATGTACGAGTTGCCTAAGTAATTCCTGTCCACGGATGGGTCGTTAAGAGCAACATTTTGTGGATCAGTAGTGCTTCGTGATAGGGAAGAACCAAGTGCAGCATCATAAGAA	77.5

SNP IDs	Desi chromosomes	Physical positions (bp)	SNPs/SSRs	Upstream 200-bp flanking sequences/Forward primers (5'-3')	Downstream 200-bp flanking sequences/Reverse primers (5'-3')	Genetic positions (cM)
CaSNP69	*Ca-desi* -chr01	6111732	(T/G)	AAAATGTATGGTGACTTCCTAGATAAGGTCATTGTGCCTAATAGGGCTGCCAGATCCAACCTGAGAACTAGATAACCAATTTGCCATTGGACTTCCTGGATAAGACAAACCAGTGCCATACGCAGGATTTCCATATGACTGGAACCACCGGATTTACCACCCATTGGAACAATGTTTGGTGACTTCTGAGGTGAAAGCAGC	ATCCAAGATAAGCTTTCTAAAGCTCAAGTAAATTCATGTACGAGTTGCCTAAGTAATTCCTGTCCACGGATGGGTCGTTAAGAGCAACATTTTGTGGATCAGTAGTGCTTCGTGATAGGGAAGAACCAAGTGCAGCATCATAAGAATATGAAGCATGGGGCCCAACATTTTGGGCTACAAATGACCCCTGAACATTTGATC	78.4
CaSNP70	*Ca-desi* -chr01	6113445	(T/C)	AGTTTATACAAGCGATTTCACTACATGCCTCTCTCATATTCTCTCATTCAATATGGTATCTAGAGCTtGGTTCGATCCTCCCTGACATGTCTTGTCACTATTCCGCTACTGAGTTCCCTAAAGTTGAGAAATATAATAGTAGTTTTTCTTTTCCGGCTACTATCATTTCCAGCCACCATTGCTGCTACCCTCTGCTCCCAC	GGAAAATATTTCCGGTGTACTCACTTCCATTGCATGCAAAATCTCTCGACCTACGCAGCCCATAGATTTTCACACCTAGAAGTAGCTCCATGCGCCGTCCACCGCCACCATGTTCATCCCCACATCTTTTGAGATAAAGAGGCGGACATTTGGAGAAAGGCATCTGTAACTTCAGTGAGAGGTGGAGTATCCCAAGAAAAG	79.6
CaSNP71	*Ca-desi* -chr01	6262893	(T/A)	TATGTTTGTAAATGAAATAGGAGGATTTAAAACTCATTAAACCTGTGAAAGCAAATCAGCAGCTACGTGAGACGGAATTGCATGTTTGTCTGCAATGACTAAAACTTCAGATGGACCAGCTGGCATGTCAATTGAAACCATAGCTTCACTATTCTGCAAAAGATTTTGAAAAAGACAAGAAAAAGAGAAAGAAGTTTTTAG	TTCAAATTATGAAATGCGATAGTGGATGTGGATACAAATCAAGAGATTCAAATATAAACACACTTGAAGTATCATTTTTGCAGCTGTAACGTATTGATTTCCAGGGCCAAAAATCTTCTCAACCTGCAAAAGTTGCCCACAAATTCACAATTAGAAATGATAGAAAAATATATGAATTATGAATACTGTTATAATTAAAGA	80.5
CaSNP72	*Ca-desi* -chr01	6262953	(A/C)	AGCTACGTGAGACGGAATTGCATGTTTGTCTGCAATGACTAAAACTTCAGATGGACCAGCTGGCATGTCAATTGAAACCATAGCTTCACTATTCTGCAAAAGATTTTGAAAAAGACAAGAAAAAGAGAAAGAAGTTTTTAGTTTCAAATTATGAAATGCGATAGTGGATGTGGATACAAATCAAGAGATTCAAATATAAAC	CACTTGAAGTATCATTTTTGCAGCTGTAACGTATTGATTTCCAGGGCCAAAAATCTTCTCAACCTGCAAAAGTTGCCCACAAATTCACAATTAGAAATGATAGAAAAATATATGAATTATGAATACTGTTATAATTAAAGAATCAAATCCTAATATTTAGGATTAGAATTAGCCTAAGAAGTGCGGACTCCGACAAAGACA	81.4
CaSNP73	*Ca-desi* -chr01	6304541	(A/G)	GGCTAGAGAAGCTGTTGCTATGAGGTCTAAGGTTCAGAAGGAAATGCTTTtGAAGGAGAAGGAAaGGAaGGAACAGGAGTTGAGGGCTTTGGCTCAGAAAGCACGGTCGGAGAGAATTGGTGTTGCTCCACCGGCTGCTGCTGCTTCGGTTGTGAGTGGTGTCGATGATGGTGGTGATATGAGAGTTGATTATGAGCATAG	GATAGGGAAAGGgATAATAGGGAAAGGGAAAGGGAAAAGAATTTTCCGAAGGAGAGTAGGGAAGAAAGAGAGGAGCGGTTGCAACGTGAGAAGATTCGTGAGGAAAGGCGAAAGGAGAGGGAGAGGGAGAGAAGGTTAGAGGCTAAGGATGCTGCCATGGGAAAGAAGAGCAAGATTACTAGAGATAGGGACCGTGATATA	82.4
CaSSR7	*Ca-desi* -chr01		(TS71)	GGTAGACGCAAAAGAGTGGG	GCCACATTGACCAGGAATG	83.6

SNP IDs	Desi chromosomes	Physical positions (bp)	SNPs/SSRs	Upstream 200-bp flanking sequences/Forward primers (5'-3')	Downstream 200-bp flanking sequences/Reverse primers (5'-3')	Genetic positions (cM)
CaSNP74	*Ca-desi* -chr01	6313759	(T/A)	TCTTACAATATGACAACACCAACTGCTACAATTTAATCCTCTGATATACAGATTCCCTGAAGATAATTTAATATTCAACAATCAATTATTAGATTAATAAATAAAATTTAGGCAGCAGAGCCTATCAGGAAGCATACGACCTCAATAAAATAAGAAACCGAACCCTAATCAGCCAATGAAGTATACAATATTTCAACTGTT	TAGGAAAAATTATGCTGCATAGAGATACAAGGGCCTGTTTGATTGAAAATTTTAACAATAGTTTTCTGTTTTCAAAAGTTAACAAAGAACAAAGATGGTCTATTGTGGACAATGACTTAAAGTCACCCAATATTCTAACACAATCAACGACAATCCTCATAAAAGTATAAGTTAACCCCCATCCCCATCTGTAAAGATGAG	84.5
CaSNP75	*Ca-desi* -chr01	6319012	(A/G)	ATCATCGAGAAAATGCAGCTCACCAATCAACCTATCCGGCGACCTTGTATCTAGTCTTGAGAGATTTTCAGCAGTCAATGAATCACCAGACCCTGGTGACCATGAAAAGTGGCTGTGTTTTGATGGAGAGAATCCAGCTACAGCTGCAACTTTTTCATCAGGACTAACTATTTCCGATGGCGACCCTTTACGTGAGGCCAC	AGGTCCTCGGCTGATACAACCAAAGCACCACCCCTCTCTGTCGAACGAACTGGTCCAGAGATAACTGGCTGCGAGCGTCCACGGATATCCTTACCTGTGTCATATTCTGGTGTAGGAGATCTTGACATACGTATGTAGTGTATGAAGACAGCCAGTAAGATTAATATGAAGAGAGCAACCACACATGACACTATAATTATT	86.9
CaSNP76	*Ca-desi* -chr01	6322174	(A/G)	TTAGCAGACCATTTAATAACTACACATAGTAAACTAAAACATAAATGCACTGAACTTAACCAAACAAAAaGAGTAAACATTATTTTTGAGAGATCTGAAAAACTTAAAACACATTATTTCAAACCAAAATCACGTCTTATAAGTACAATGTAGACTGCAGCAGAAAACCCTACCTTCACATATCAAACTTAAGAACACAAC	CAAAGATTAAAACTTTAAAAaCTCATAGAGTTGAAGATTTGAAGTACAGAGAAGCAGCATTTTGGCTACAACTACTCATGAAGAAAAGGGAAATTAGTACCCAATTTGCATCCATGCTGCAATAAAGAGGAAGGGACCCAATAACTTTTTTtGGCCCTAAAAAGCTTTGATGGAAGGAGTGAATTCTTTTTCCAACCCAAA	88.9
CaSNP77	*Ca-desi* -chr01	6322202	(G/T)	GTAAACTAAAACATAAATGCACTGAACTTAACCAAACAAAAaGAGTAAACATTATTTTTGAGAGATCTGAAAAACTTAAAACACATTATTTCAAACCAAAATCACGTCTTATAAGTACAATGTAGACTGCAGCAGAAAACCCTACCTTCACATATCAAACTTAAGAACACAACACAAAGATTAAAACTTTAAAAaCTCATA	AGTTGAAGATTTGAAGTACAGAGAAGCAGCATTTTGGCTACAACTACTCATGAAGAAAAGGGAAATTAGTACCCAATTTGCATCCATGCTGCAATAAAGAGGAAGGGACCCAATAACTTTTTTtGGCCCTAAAAAGCTTTGATGGAAGGAGTGAATTCTTTTTCCAACCCAAAAAAAaTAGTAACAAAAATTTCTCTTTTT	89.5
CaSNP78	*Ca-desi* -chr01	6333418	(G/A)	AACCAGACCTTGTTCAGACTGCGGGTTGGCACGCTTAATTGCAGCAGGGACACCATCATCAACCTCTCCTTTATACACCTTACCAAAGCCTCCAACTCCGATGATCAGACTCTCGTCGAAACTATTTGTTGCAGCATTAATTTCGGCCAATGTAAACCGCTTACCAGCTCTGGTTGAAGCCACAGATCCATATAACTTTTG	GTTCCAGCCGATCCCTTGGCACCTCCCACAGTGCTGTTTACAGCAGCCCCATACAGGAATATCGGTCGCCAACCCGGAGAGTTGTTTTTGGTATCACTTGATTCTTTTCTCCTTTTAAAGAAACAGAAAATAAAGACACCAATACATGCCATTATAGCTACAGAAGCTATACCTGCTCCTACTCCAATCCAAACAACTCTT	90.5

SNP IDs	Desi chromosomes	Physical positions (bp)	SNPs/SSRs	Upstream 200-bp flanking sequences/Forward primers (5'-3')	Downstream 200-bp flanking sequences/Reverse primers (5'-3')	Genetic positions (cM)
CaSNP79	*Ca-desi* -chr01	6341213	(A/C)	ATGCATTTGCTCCTTGTATTGTTCAATTTCCTTTGCTG<br>CTGCAACAAGGTTACCCTTAGTAACATCCAACTCTTT<br>ACTGTTCTCCACAATCCATTTATGTTGACGATCTGTCT<br>TAGTTCTTAAACTGTCAAGCTGTTCCGAGGCAGATTC<br>CATCTTACACTTTTCTTGTTCCAAAGCATCAGTTGCCG<br>CTTGGATTATATT	TCTTTTTCTTCCACTATTGATTTCAATTTAAGTATTTCAC<br>ATTTAAGTTTATCATTCTCAGCAGCTTTGAATTTTAGTG<br>CTTCATCCGCATCTACCAAAGCTTCTTTAAATATAATTTG<br>ATTAATATCCAGTAGCCCTTGCATCATAATTGACTCCAT<br>ATTTGCTTCTTCTATTTCCAACCCATTGGAAGCTTGAGC<br>ACTG	92.4
CaSNP80	*Ca-desi* -chr01	6341237	(C/T)	AATTTCCTTTGCTGCTGCAACAAGGTTACCCTTAGTA<br>ACATCCAACTCTTTACTGTTCTCCACAATCCATTTATG<br>TTGACGATCTGTCTTAGTTCTTAAACTGTCAAGCTGTT<br>CCGAGGCAGATTCCATCTTACACTTTTCTTGTTCCAAA<br>GCATCAGTTGCCGCTTGGATTATATTATCTTTTTCTTC<br>CACTATTGATTT	AATTTAAGTATTTCACATTTAAGTTTATCATTCTCAGCA<br>GCTTTGAATTTTAGTGCTTCATCCGCATCTACCAAAGCT<br>TCTTTAAATATAATTTGATTAATATCCAGTAGCCCTTGC<br>ATCATAATTGACTCCATATTTGCTTCTTCTATTTCCAACC<br>CATTGGAAGCTTGAGCACTGTCGATAGTTTCTTTCAATA<br>TTATA	93.5
CaSNP81	*Ca-desi* -chr01	8173005	(T/C)	TTCGACTAGATTAGATTAGAGGAAACTGTAAAGAAA<br>AAGTGAAAGTGAATGGAATACCGGATACGATAGAA<br>GGGATTGGAAGGAGTGAAGAGAAGCGCGAATGGCG<br>TGTAAGAGTTGGACCCGTTGAGACGGTGTTGGGGGA<br>GAGGAACCGAGAAGCGCTGATTCCAAGGTGGCTAG<br>AAGCTGCTTCGGAGAGACCATTTG	GCCGCCGGTGTGATGATGAAATGAAACCCTACGAGTC<br>ACAACACAACTGAGAGAGAGAACACAGGTACTAGGGC<br>GGGAAGGAGGAAGTGAGTAAATTTTGAGTCCACAAAA<br>ACAAATTTGCAGCTCAAAGTAGTAGCGCCCTGCTGGTG<br>ACGTCTTAAACCCTATCAAACATAAAGTACAAAACCCCT<br>CCACTTGGTTTTC	94.5
CaSNP82	*Ca-desi* -chr01	8871945	(C/T)	AAGGGAGTAGAAAAATGTTGCAGGGTAGTAATGCG<br>GTGGCGGAAGCAAATAGTGATGATGGTGGTGTGAC<br>GAGAGAGGGTATGTACAAAGGAACGGGAGAAGAG<br>ATGGGTATGTCAAGTGATGCAGCAAGAGAGGGTCCA<br>ATAAAAGCAGAGGAAGAAGGAGATATGGTGAGAGA<br>TACTGCAAAGGATAGCATGGATGGAG	TTGGATAGCAGCTACAAACAAGGTTCGAGATTGTCACA<br>AGGATGAAGGGCAACGATAATTATATAAGGAGGAAAC<br>ATCTTATAATTCCACAATGTTTTATTCATCAAAATGTGTC<br>ATCACTTCTTTATGTAATGCCTACTTAATTTCAAACCACT<br>TGCATAATTATGGGTCGTTATGCTTTTCCATTTGCCTATT<br>TACAAC	95.6
CaSNP83	*Ca-desi* -chr01	10106781	(G/C)	TTtATCAAACCTACAAAATAAATAAaCCTAAAAAAAa<br>GGATATAACCGTCATTTCATATAGACGAAACCGAAGT<br>GGTTTCAACCCTAATTATAAATATATAAGACTACCTTC<br>ATTTTTACAGTGTTCCACTTGCAGCGCCTCGTGATATC<br>TCTCTCTGGTGTCAAAACCCTAACATCCAGATCTCGCT<br>ACTCTTCCATTAC	ATCAAGTAAGTTTGCGACTTTTTCACAATCAATTTCAAT<br>TCCATCTATGTCTCATTGATTCTAACCATTTTTGTTTGCT<br>AATTTTTTGAATCAGATATGAAGATCATCGCTGCTTACT<br>TGTTGTCTGTATTGGGAGGTAACTCATCCCCTTCAGCCG<br>ATGATGTTAAAAAAaTTCTCCACTCAGGTTCGTAATTTT<br>AATTC	96.4

SNP IDs	Desi chromosomes	Physical positions (bp)	SNPs/SSRs	Upstream 200-bp flanking sequences/Forward primers (5'-3')	Downstream 200-bp flanking sequences/Reverse primers (5'-3')	Genetic positions (cM)
CaSNP84	*Ca-desi* -chr01	11569811	(A/C)	TCACCGACCATACCGAAGAACTTGGCTTGAGAACAGGCTGGAGGACAACCTTCCAACTTGGGAATGCTTGAAAGGCATGATTCAGCATCCTCAAGTTGATGGAGTTTCAAATAAGCTTCTGCTTTACAAGCCACAAGCTGCAAGTAACAGTCCAACAAGTAAGACAACACATACCATTGGAAAATCCAAAATTTTAGTCTC	cTTCATCAATTAAGAAGTACTACCTGAGGTGAGAAGTCTGCTCCAATCGCAACAGCAGCCTCTACTTCCTGGATTGCCCTTTTCCAATCACCGATCTTCCGCACATCCGCACATCGGTTCACATGCTTCTCCAACAATAACAGCTTCTGCTCTTCCAATTGATCGTTCTGCAAACCCGCATAATATAGGTGGCGACGCGAG	97.3
CaSSR8	*Ca-desi* -chr01		(TR55)	TTACTCAACCATAATAATAATAATAAT	CTCTTCAATCTTCACTTATTCAT	98.5
CaSNP85	*Ca-desi* -chr01	12159674	(A/C)	CGTTTATAACTGAGCAACAACAATAGCTTATTGGTAAAAAAaCAATCAACAAAAGAATAATTAAAAGGGTGGATGTGGTGATCATAACACAACAAAAAGGTGCAAGTAATCATAACATAAAACAAACGGGTGCATGTGATAGAATGCATACAGTTCATTTGCAGCATACAGTTGTGCTTCATCTTTAGGCATGCTGCAATa	aaGATCAAATTATAATTACTACTATTAGTATTATAAATGCAAGCATATGTGAAAATGAATATGATTGGTTTTCAAAAAAGCGCACCTGTAAAATATGTAAAACAGTGTTTCCAATGTAAACTTTGCAAAATAACCTTGCTGCACCACACAaGAATTAGCCCACATAAAAaGCAGAAAATAAGGACAtAGaaataTAAAGCT	99.6
CaSNP86	*Ca-desi* -chr01	12163294	(C/A)	AAAGGCTTGATGTAGGTTGCTGAACCCCTCCAGTTGGCATGCTATTTATTGTTGAGTTTCTTGATGTAAGTGTACTGGGCATGTTGGGAACGTTGAAGCTTCCATGCATGTTGTGCAACCCCTGAATACCACCTGTAGcAAAGAGCTAGCTATTATGCAACTAAAGGAGAGAAGAAAATGAATTGACTCCAGCAAAATATT	TCACCAGTGTGATGATAAATTGGGGAGGCTGCACCAGACTGACTCGAGAATGATGTAGTGAAAGAACGCCCAGCACCGTCTGGAAGATTTGAAGTAGAACCGTTCAGAGAAGACTGCAAACAGAACTCTGTTATAATTTACATCAATTGTGAAAGGCGAAAAGCACAAATCAATATCCGACAAAAATTATAATGATGATAT	100.1
CaSNP87	*Ca-desi* -chr01	14093206	(T/A)	TTTGCTAACTTTGCCAACTTATGGGCCTCTCTCTACATTATTTTGTCTACCAATGAAACTGTCAGAAGCATGTCTAAAACTAAaCATCAGATCATTACAATCCAATTACACTTCTATGCTTTAGATATTTTGTtCGgCTGCCAaTTGAAGGCACCAGCACACACCAAGAGCTTCTGCCGTATACGAATCTGAGGCAATGCA	TCTTTTCTGCAAGCTGCCAAAGAAACAACACCCATTGGATCTTTAAAAaCACATCCCCACGTGGTAGGGCCATCCAGACAGCCCCcTCATCAACCTGTGCAGTAAAATTAGCAGTGACAGTTCTGATTTATGGTATGCTTTCCTGCAGAATGTCATTCCCCTGCTTGGTTAAAATTTGAGACAAAATCACAAGCACTCCTA	100.9
CaSNP88	*Ca-desi* -chr01	14114903	(A/C)	CCATCCCCCTTGGCCCTTTTCGGCCCTTTGACTACTAATGATAAGAGATGGTGATGTTAATTATTAATATCAATAATAATAATAAGAAAAAGATAAAAGAATCACATATATGCAGCCGAATACAACCAATACCAATACCCTCTTAAGAATAAGAGAATTATTATATATCTGGCGAGCGGGTTTTGGCTTTATCATCTTGAT	TCCTCGTTATCTTTATCTGCGATTGCCATTGTCAATGCTAATGGGCGCGCGCTCTAATTCAATTGTGCGCGTACCTGCAGCATATAATTATAATGTCAAATCCTTAGTTGTTTGGATATTATTACTTTCATGTGCAAGCATGTCAATGTCATATTCAGATGTTGAGGCGTTGTTGAAGTTCAAGGATTCATTAACAAATGT	101.8
**Markers localized at the major seed weight QTL interval (*CaqSW1.1*) are highlighted with red colour**						
