# Supplementary material for: Deploying QTL-seq for rapid delineation of a potential candidate gene underlying major trait-associated QTL in chickpea
Source: DNA Res. 2015 Apr 27;22(3):193–203. doi: 10.1093/dnares/dsv004 (PMC4463844; doi:10.1093/dnares/dsv004)
Supplement: Supplementary Data [file supp_dsv004_dsv004supp_table2.pdf]

Table S2: Structural and functional annotation of six protein-coding genes localized at the 35 kb genomic interval of a major SW QTL (*CaqSW1.1*)

***Desi* gene IDs**	**Chromosome**	**Start physical positions (bp)**	**End physical positions (bp)**	**Directions**	**Putative functions**
Ca_00071	*Ca-desi*-chr01	836065	839806	plus	COP9 signalosome complex subunit 8
Ca_00072	*Ca-desi*-chr01	840671	841007	minus	Putative uncharacterized protein
Ca_00073	*Ca-desi*-chr01	844344	845613	minus	Fasciclin-like arabinogalactan protein 19
Ca_00074	*Ca-desi*-chr01	847138	848472	plus	Vinorine synthase
Ca_00075	*Ca-desi*-chr01	849660	852957	minus	Bidirectional sugar transporter N3
Ca_00076	*Ca-desi*-chr01	863532	951771	plus	Protein FAM135A
